# Pervasive and dynamic protein binding sites of the mRNA transcriptome in *Saccharomyces cerevisiae*

**DOI:** 10.1186/gb-2013-14-2-r13

**Published:** 2013-02-14

**Authors:** Mallory A Freeberg, Ting Han, James J Moresco, Andy Kong, Yu-Cheng Yang, Zhi John Lu, John R Yates, John K Kim

**Affiliations:** 1 Life Sciences Institute, Department of Human Genetics, University of Michigan, Ann Arbor, MI 48109-2216, USA; 2 Department of Computational Medicine and Bioinformatics, University of Michigan, Ann Arbor, MI 48109-2216, USA; 3 Department of Cell and Developmental Biology, University of Michigan, Ann Arbor, MI 48109-2216, USA; 4 Department of Chemical Physiology, The Scripps Research Institute, La Jolla, CA 92037, USA; 5 MOE Key Lab of Bioinformatics, School of Life Sciences, Tsinghua University, Beijing, China 10008

## Abstract

**Background:**

Protein-RNA interactions are integral components of nearly every aspect of biology, including regulation of gene expression, assembly of cellular architectures, and pathogenesis of human diseases. However, studies in the past few decades have only uncovered a small fraction of the vast landscape of the protein-RNA interactome in any organism, and even less is known about the dynamics of protein-RNA interactions under changing developmental and environmental conditions.

**Results:**

Here, we describe the gPAR-CLIP (global photoactivatable-ribonucleoside-enhanced crosslinking and immunopurification) approach for capturing regions of the untranslated, polyadenylated transcriptome bound by RNA-binding proteins (RBPs) in budding yeast. We report over 13,000 RBP crosslinking sites in untranslated regions (UTRs) covering 72% of protein-coding transcripts encoded in the genome, confirming 3' UTRs as major sites for RBP interaction. Comparative genomic analyses reveal that RBP crosslinking sites are highly conserved, and RNA folding predictions indicate that secondary structural elements are constrained by protein binding and may serve as generalizable modes of RNA recognition. Finally, 38% of 3' UTR crosslinking sites show changes in RBP occupancy upon glucose or nitrogen deprivation, with major impacts on metabolic pathways as well as mitochondrial and ribosomal gene expression.

**Conclusions:**

Our study offers an unprecedented view of the pervasiveness and dynamics of protein-RNA interactions *in vivo*.

## Background

A diverse and expanding repertoire of RNA-binding proteins (RBPs) ensures faithful expression and function of substrate mRNAs [1-3]. Many RNAs are organized by RBPs and other protein co-factors into higher-order ribonucleoprotein (RNP) assemblies that fulfill critical functions in storage, transport, inheritance, and degradation of RNA [4,5]. For example, over 70% of mRNAs in *Drosophila *embryos are localized to distinct organelles, compartments, and membrane interfaces, providing a means for directing local translation and regulating cellular architectures and functions [6]. RNA and RBPs can also reversibly aggregate into granules to allow RNA storage and decay in response to stimuli [7,8]. These and many other processes are driven by large, complex networks of protein-RNA interactions that provide specificity in gene regulation and fidelity in RNP assembly. Despite important insights regarding the necessity of RNA regulation for cellular functions, the RBP-RNA interactome and its response to changing cellular conditions have yet to be fully elucidated.

Studies of RBP-RNA interactions have historically relied on the identification of target transcripts bound by individual RBPs. *In vitro *selection of RNA sequences that bind RBPs with high affinity (systematic evolution of ligands by exponential enrichment, SELEX) can identify primary sequence recognition elements. For example, Nova proteins, which regulate mRNA splicing in neurons, recognize the RNA consensus sequence YCAY [9], and Y box-binding protein-1, a member of the cold shock/Y box domain protein family, recognizes a CAYC RNA motif [10]. Yet these and other primary sequence elements identified *in vitro *are generally short and degenerate and appear too frequently in the transcriptome to be useful for *in silico *target identification. Microarray profiling of transcripts that co-purify with interacting proteins (RIP-Chip) has been widely used to detect transcripts stably associated with RBPs, such as mRNAs bound by translational components HuB, eIF-4E, and PABP in P19 embryonal carcinoma stem cells [11]. Similarly, RIP-Chip experiments have identified mRNAs associated with 40 yeast RBPs and uncovered a set of potential RBP recognition motifs [12,13], some of which were validated *in vitro *using SELEX [14]. Although capable of identifying mRNA targets for select RBPs, RIP-Chip is prone to artifacts, including RBP-RNA dissociation and re-association after cell lysis [15], isolation of non-specific RNAs, and indirect binding through other co-purified RBPs [16]. In addition, RIP-Chip cannot detect transient interactions or resolve the exact RBP binding sites on identified transcripts.

To identify transcriptome-wide footprints of RBPs *in vivo*, UV crosslinking has been coupled with immunopurification of RBPs (CLIP) [17,18]. CLIP takes advantage of the photoreactivity of RNA bases, most often pyrimidines, with interacting amino acid side chains upon 254 nm UV irradiation [19]. The formation of covalent linkages allows stringent purification of RBP-RNA complexes and subsequent identification of crosslinked RNA fragments via cDNA sequencing. Recently, a modified CLIP technique, PAR-CLIP (photoactivatable-ribonucleoside-enhanced CLIP), has been introduced in which photoactivatable-ribonucleoside analogs are incorporated into the transcriptome in live cells to enable efficient crosslinking using 365 nm UV irradiation [20]. Recent studies employing CLIP in mouse brain [21] and *Caenorhabditis elegans *[22] and PAR-CLIP in human embryonic kidney cells [20] have successfully decoded *in vivo *microRNA-mRNA interactions by identifying RNAs bound to Argonaute, a main component of the microRNA-induced silencing complex. PAR-CLIP has also been implemented to elucidate the regulatory mechanisms of human antigen R (HuR) protein, which stabilizes gene expression by binding to AU-rich elements [23,24], and to identify the transcriptome-wide distribution of non-poly(A) termination factors in yeast [25]. In addition to enabling efficient crosslinking, PAR-CLIP generates frequent and non-random nucleotide substitutions at crosslinking sites to reveal specific RBP-RNA contact sites with nucleotide resolution.

Until recently, CLIP and PAR-CLIP have been limited to investigation of individual RBPs. Two recent studies introduced the use of photoactivatable-ribonucleoside-enhanced UV crosslinking with oligo(dT) pull-down of mRNAs followed by tandem mass spectrometry to globally identify mRNA-binding proteins in human cell lines [26,27]. In addition to identifying known RBPs, these studies identified 315 [26] and 245 [27] novel RBPs that lack canonical RNA-binding domains and functional annotation as RNA-binding proteins. Castello *et al*. [26] found that RBP amino acid sequences are more disordered than those of non-RBPs and identified potential new classes of RNA-binding domains. Baltz *et al*. [27] additionally captured and sequenced protein-bound mRNAs, providing a transcriptome-wide map of potential *cis*-regulatory elements.

Despite recent advances towards understanding global RBP-RNA interactions, the dynamic nature of these associations *in vivo *and the general principles driving these associations remain unexplored. Here, we adapt the PAR-CLIP technique to map all RBP binding sites across the yeast non-translating mRNAs in different environmental conditions, a method we call global PAR-CLIP (gPAR-CLIP). The comprehensive identification of RBP-RNA crosslinked sites visualized by gPAR-CLIP allows us to derive general properties of RBP-RNA interactions *in vivo*. Additionally, we compared RBP-RNA crosslinked sites in rapidly proliferating versus stress-treated cells and observed large-scale changes in RBP-RNA interactions, providing a starting point for dissecting the network of post-transcriptional gene regulatory mechanisms underlying stress response.

## Results

### gPAR-CLIP identifies transcriptome-wide RBP crosslinking sites

**Figure 1:**
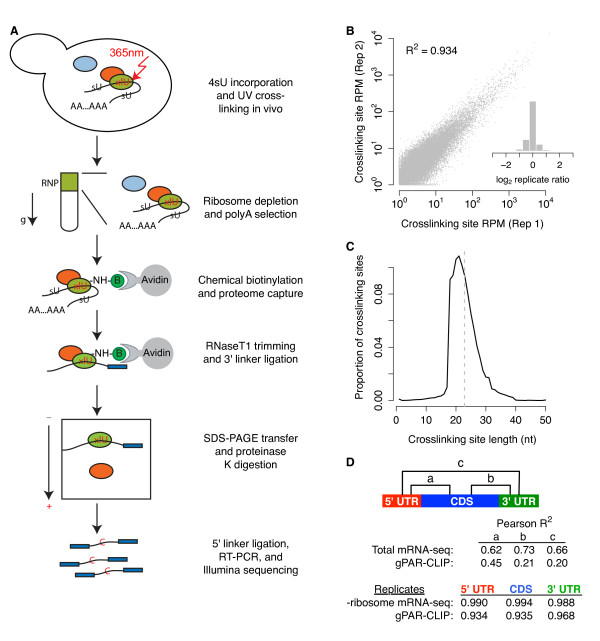
**gPAR-CLIP identifies transcriptome-wide RBP crosslinking sites**. **(a) **Schematic of the gPAR-CLIP protocol. **(b) **Reproducibility of crosslinking sites generated from replicate gPAR-CLIP libraries prepared from yeast grown in synthetic defined media (abbreviated as WT gPAR-CLIP hereafter). Pearson correlation coefficient is indicated. Inset: distribution of log_2 _crosslinking site RPM ratios between replicates. Replicate error σ = 1.3-fold. **(c) **Length distribution of crosslinking sites in WT gPAR-CLIP libraries. Dotted line: average crosslinking site length of 23 nucleotides. **(d) **Pearson correlation coefficients of total mRNA-seq and gPAR-CLIP read coverage between 5' UTR, CDS, and 3' UTR regions as well as correlation coefficients of ribosome depleted (-ribosome) mRNA-seq and gPAR-CLIP read coverage between replicate WT libraries.

To construct a global map of RBP binding sites on the transcriptome *in vivo*, we combined PAR-CLIP with high-throughput sequencing (Figure 1a; Materials and methods). Briefly, we metabolically incorporated the photoactivatable-nucleobase analog 4-thiouracil (4sU) in growing yeast and used UV irradiation to crosslink 4sU to juxtaposed proteins, 'freezing' protein-RNA interactions *in vivo*. Next, we implemented three biochemical strategies to capture RNA regions bound by the proteome: (i) sucrose gradient centrifugation to reduce ribosome abundance; (ii) oligo(dT) selection to deplete abundant structural non-coding RNAs (for example, rRNAs); and (iii) chemical biotinylation of proteins. We exploited the high-affinity streptavidin-biotin interaction to purify all biotin-protein-RNA complexes with high efficiency and stringency. After trimming unbound RNA, RBP-protected fragments were ligated to linkers, converted to cDNAs, and subjected to Illumina high-throughput sequencing (Materials and methods). We term this global PAR-CLIP method 'gPAR-CLIP'. There are two caveats associated with our gPAR-CLIP protocol. First, during crosslinking (approximately 5 minutes), the cells were in nutrient-free buffer and incubated on ice, which could trigger changes in RBP binding. Second, we limited our analysis to mRNAs from the top of the sucrose gradient, which mostly consist of non-translating mRNAs, so our conclusions apply to non-translating mRNAs.

To define the dynamic landscape of RBP-RNA interactions, we constructed duplicate gPAR-CLIP and mRNA-seq libraries, including incorporation of 4sU, for the wild-type *Saccharomyces cerevisiae *strain cultured in complete media or subjected to glucose or nitrogen starvation for 2 hours. An average of 10 million reads were sequenced from each gPAR-CLIP library. Of the 72% of reads that mapped uniquely to the genome, over 70% contained one or two T-to-C conversion events, the signature substitution induced by 4sU crosslinking (Additional file 1). From overlapping reads, we derived clusters representing RNA regions crosslinked to proteins (Additional files 2 and 3; Materials and methods). Crosslinking site read coverage was normalized to mRNA expression levels calculated as reads per million mapped reads per kilobase of transcript (RPKM). Because our approach captures protein-RNA interactions for potentially all RBPs, we cannot rule out the possibility that some clusters are located proximally to true RBP-binding sites [28]; therefore, we refer to our gPAR-CLIP read clusters as 'crosslinking sites'. We empirically assigned a false discovery rate (FDR) to each crosslinking site by deriving clusters from mRNA-seq reads with one or two T-to-C mismatches representing sequencing error and comparing the T-to-C conversion rate of these clusters to those derived from gPAR-CLIP reads (Materials and methods). Using a 1% FDR threshold, we reproducibly identified 80,883 crosslinking sites that are, on average, 23 nucleotides long (Figure 1b,c): 65,992 in protein-coding sequences (CDSs), 4,508 in 5' UTRs, 8,525 in 3' UTRs, and 818 in introns (Additional files 3 and 4). Of 6,717 annotated protein-coding transcripts, 6,228 (93%) have at least one crosslinking site. Because CDS crosslinking sites exhibited three-nucleotide periodicity, a hallmark of ribosome binding (Additional file 5), we separately analyzed CDS, 5' UTR, and 3' UTR crosslinking sites. We observed high correlation between gPAR-CLIP read coverage of 5' UTRs and CDSs (Pearson correlation coefficient, R^2 ^= 0.45), supporting a prominent role for 5' UTRs in translational regulation (Figure 1d). However, gPAR-CLIP read coverage of 3' UTRs correlated poorly with both 5' UTRs (R^2 ^= 0.20) and CDS (R^2 ^= 0.21), suggesting a greater role in post-transcriptional regulation. As expected, correlation between total mRNA-seq read coverage across all transcript regions was approximately equal (Figure 1d; Additional file 6). We also observed very high correlation of gPAR-CLIP and mRNA-seq read coverage between replicates over each genic region (Figure 1d), reflecting low technical variation between replicates.

### gPAR-CLIP captures known and novel crosslinking sites

**Figure 2:**
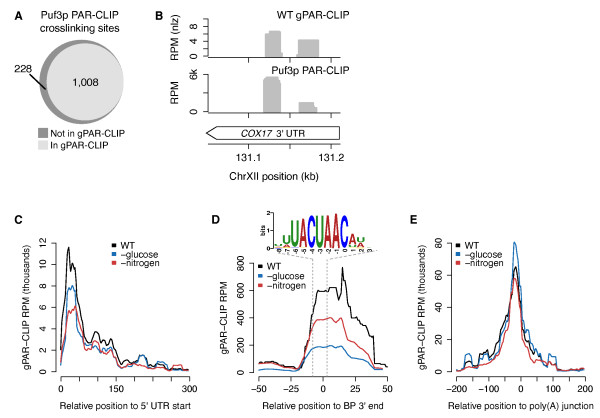
**gPAR-CLIP captures known RBP crosslinking signatures**. **(a) **Overlap of crosslinking sites identified in Puf3p PAR-CLIP and wild-type (WT) gPAR-CLIP. Puf3p PAR-CLIP crosslinking sites with >1% T-to-C conversion rate (Additional file 8) were considered captured by gPAR-CLIP if at least 50% of their nucleotides overlapped with a WT gPAR-CLIP crosslinking site with FDR <1%. **(b) **Identification of known Puf3p binding sites on *COX17 *mRNA in WT gPAR-CLIP and Puf3p PAR-CLIP. **(c-e) **Aggregate gPAR-CLIP crosslinking site coverage of the first 300 nucleotides of 2,626 annotated 5' UTRs (c), 51 annotated ribosomal gene introns centered at the branch point (BP) 3' end (d), and 4,241 3' UTRs centered on the poly(A) junction (e).

To assess the performance of gPAR-CLIP in capturing known RBP-RNA interactions, we evaluated its ability to identify binding sites of Puf3p, a Pumilio-family RBP, which we derived from conventional PAR-CLIP using a strain expressing a TAP-tagged Puf3p fusion protein. Of the 1,236 Puf3p binding sites confidently identified by PAR-CLIP, 1,008 (82%) were also captured by gPAR-CLIP (Figure 2a; Additional file 7); for example, the two functionally validated Puf3p binding sites in the *COX17 *3' UTR were identified by both Puf3p PAR-CLIP and gPAR-CLIP (Figure 2b) [29,30]. It is possible that other Puf proteins with similar RNA recognition motifs are binding at these sites in our gPAR-CLIP libraries [31], reflecting that our protocol does not distinguish the RBPs associated with each crosslinking site. From our Puf3p PAR-CLIP library, we also identified 560 novel Puf3p mRNA targets harboring a binding site containing a Puf3p recognition motif (Additional files 8 and 9). Given the high recovery of Puf3p sites by gPAR-CLIP, we conclude that gPAR-CLIP faithfully captures binding sites of a known RBP.

We next examined our data for general RBP-RNA interaction signatures related to mRNA maturation and translational regulation. gPAR-CLIP read coverage of 5' UTRs peaked within 75 nucleotides downstream of annotated transcription start sites but was reduced when yeast were grown in media lacking glucose or nitrogen (Figure 2c). This coverage likely reflects RBP-RNA interactions involved in translation initiation, and the decrease in coverage is consistent with decreased translation initiation that occurs during cellular stress [32-34]. gPAR-CLIP also effectively captured the spliceosome binding pattern by identifying intronic RBP crosslinking sites clustering 3' of the lariat branch point (BP) bound by the U2 snRNP (Figure 2d). These crosslinking sites contain the canonical BP-binding protein recognition sequence UACUAAC [35,36]. Consistent with stress-induced transcriptional repression of ribosomal subunits [37], which account for 18% of all protein-coding genes with introns, gPAR-CLIP read coverage at the lariat BPs of ribosome-encoding mRNA introns decreased upon glucose and nitrogen deprivation. Finally, a strong RBP crosslinking signature was identified approximately 20 nucleotides upstream of the most prominent poly(A) junction site identified in each 3' UTR (Figure 2e), consistent with interactions with the polyadenylation complex [38]. Taken together, these results indicate that gPAR-CLIP faithfully captures diverse RBP-RNA interactions along the discrete anatomy of mRNAs.

### RBP crosslinking sites exhibit global conservation in both primary sequences and secondary structures

**Figure 3:**
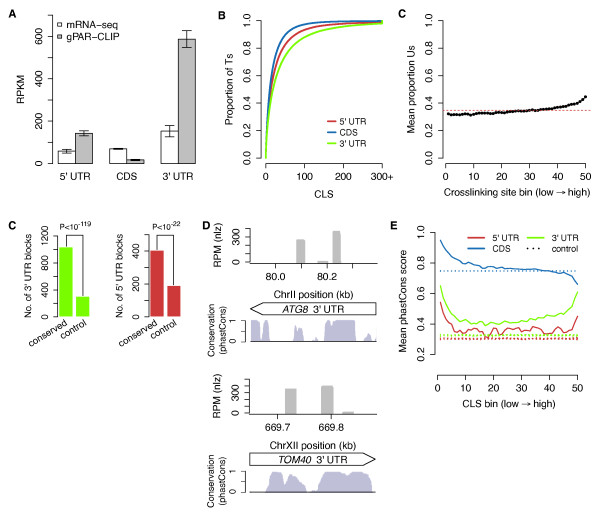
**RBP crosslinking sites exhibit global sequence conservation**. **(a) **Average ribosome depleted mRNA-seq and gPAR-CLIP read distributions across 5' UTR, CDS, and 3' UTR regions for all libraries. Error bar: 1 standard deviation. RPKM: reads per million mapped reads per kilobase. **(b) **Cumulative distribution of CLS values from wild-type (WT) libraries. **(c) **Proportion of Ts in crosslinking site binned by crosslinking site coverage (reads per million mapped reads (RPM)). The dashed red line indicates average T content of all crosslinking sites. **(d) **Number of conserved blocks in 3' and 5' UTRs overlapping 100% with WT gPAR-CLIP crosslinking sites (χ^2 ^*P*-values indicated). Control blocks were randomly generated within 3' and 5' UTRs to match the number and size of conserved blocks. **(e) **Two major gPAR-CLIP crosslinking sites in *ATG8 *3' UTR (top) and *TOM40 *3' UTR (bottom) overlapping conserved blocks. **(f) **Mean phastCons scores for Ts ranked and binned by CLS. Control lines represent mean phastCons scores of randomly ranked and binned Ts with no CLS, repeated ten times.

Compared to mRNA-seq reads, which were equally distributed among 5' and 3' UTRs and CDSs, gPAR-CLIP reads were 4-fold enriched in 3' UTRs, 2.5-fold enriched in 5' UTRs, and 4-fold depleted in CDSs compared to mRNA-seq reads (Figure 3a). To examine RBP binding activity at nucleotide resolution, we calculated a crosslinking score (CLS) for each T in the genome (U in the transcriptome) as the ratio of gPAR-CLIP reads containing one or two T-to-C conversion events to mRNA-seq reads to normalize for variable mRNA abundance (Additional file 10; Materials and methods). There were 378,247 Ts (12.7% of transcriptomic Us) assigned a CLS: high CLS values indicate high crosslinking efficiency and strong RBP-RNA interactions; low CLS values indicate low crosslinking efficiency or weak/transient RBP-RNA interactions. Consistent with the distribution of gPAR-CLIP reads, CLS values were highest in 3' UTRs followed by 5' UTRs and CDSs (Figure 3b; Additional file 11). These observations support 3' UTRs as the primary sites for RBP-RNA interactions for non-translating mRNAs. To determine if enrichment of gPAR-CLIP reads on UTRs was biased because of the U-richness of UTRs, we compared the proportion of Us in each crosslinking site to its coverage in gPAR-CLIP and observed only a weak positive correlation, which by itself cannot account for the four-fold enrichment of gPAR-CLIP reads on UTRs (Figure 3c).

A previous comparative analysis of seven *Saccharomyces *genomes revealed that approximately 14% of evolutionarily constrained bases lie outside protein-coding regions, often located in UTRs [39]. These conserved regions could represent functional elements interacting with *cis*-acting factors. We found direct evidence of RBPs crosslinking to 35% of conserved sequence blocks in UTRs as defined by phastCons, a score representing the likelihood that a base falls in a conserved element (Figure 3d): 405 of 1,549 5' UTR blocks (26%) and 1,036 of 2,536 3' UTR blocks (41%) completely overlap with at least one RBP crosslinking site, which is significantly higher than randomly defined control blocks (χ^2 ^test, *P *< 10^-119 ^for 3' UTR and *P *< 10^-22 ^for 5' UTR).

At the gene level, *ATG8*, a key autophagy gene, contains two major crosslinking sites that overlap with conserved sequence blocks in its 3' UTR (Figure 3e, top; Additional file 11). Similarly, *TOM40*, which encodes a translocase that mediates import of mitochondria-localized proteins into the mitochondria, contains two major 3' UTR crosslinking sites in regions with high local conservation (Figure 3e, bottom; Additional file 11). To further elucidate the connection between RBP binding and conservation, we binned Ts by CLS values and observed that Ts in all 3' and 5' UTR bins, as well as the majority of CDS (78%) bins, were more conserved than randomly binned Ts, suggesting that RBP crosslinking sites are under purifying selection (Figure 3f; Additional file 11).

**Figure 4:**
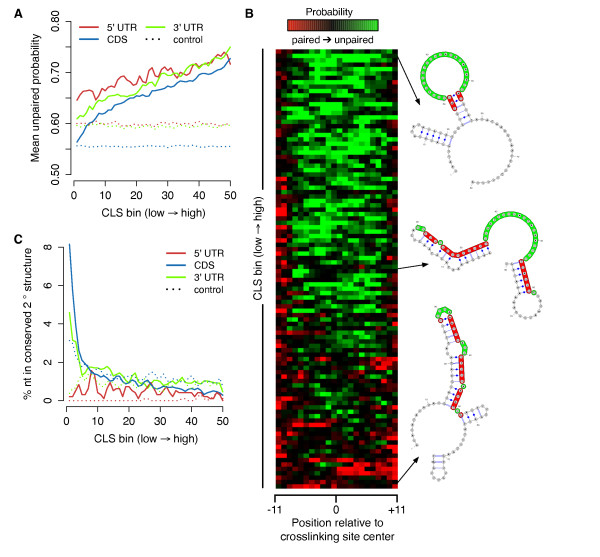
**RBP crosslinking sites share global structural characteristics**. **(a) **Mean unpaired probability scores for Ts ranked and binned by CLSs. Control lines represent mean unpaired probability of randomly ranked and binned Ts with no CLS. Pearson correlation coefficients: 5' UTR R^2 ^= 0.933, CDS R^2 ^= 0.976, 3' UTR R^2 ^= 0.986. **(b) **Crosslinking site pairedness visualized as a heatmap. Columns represent nucleotide positions within crosslinking sites. Rows represent average unpaired probability for 100 crosslinking sites in that bin. Select secondary structure predictions from low, middle, and high CLS regions are indicated with the crosslinking site colored. **(c) **Percentage of Ts ranked and binned by CLSs in conserved secondary structural elements as defined by RNAz [43,44]. Control lines represent percentage of randomly ranked and binned Ts with no CLS in conserved secondary structural elements.

Unexpectedly, 3' and 5' UTR nucleotides in the lowest CLS bins exhibited extremely high conservation. Since a low CLS can indicate inefficient RNA capture, and gPAR-CLIP inefficiently captures highly structured, double-stranded RNA (see Discussion), we hypothesized that low CLS/high conservation bins represent conserved, secondary structure motifs recognized by RBPs. For example, She2p binds a distinct stem-loop structure in several bud-localized mRNAs [40,41], including *ASH1*, for which the She2p 3' UTR recognition element is weakly represented in our gPAR-CLIP dataset. To determine if Ts with low CLS values are located in RNA regions with a high degree of secondary structure, we computed the probability of each T being unpaired using RNAplfold, a local thermodynamic folding algorithm [42]. We observed that Ts with low CLS values exhibited low unpaired probabilities, suggesting they are more likely to exist in double-stranded structures (Figure 4a; Additional file 12). Additionally, a strong, positive correlation between unpaired probability and CLS values indicates that unpaired regions crosslink more strongly to RBPs. To probe RNA secondary structures more accurately, we extended the boundaries of each crosslinking site to span 80 nucleotides and calculated the most thermodynamically stable secondary structure. Consistent with the per nucleotide analysis, crosslinking sites with low CLS values formed predominantly double-stranded RNA structures (Figure 4b; Additional file 12).

Secondary structures tolerate substitutions that preserve base pairing in stem regions, a characteristic known as covariance. To identify conserved and thermodynamically stable RNA secondary structures using a covariance model, the seven yeast genomes were scanned with RNAz [43,44], and a small set of potential structural elements was identified: 843 in CDS, 25 in 5' UTRs, and 51 in 3' UTRs. Among Ts assigned a CLS, those with the lowest CLS values in CDS and 3' UTRs were preferentially located in conserved, structural elements compared to control elements (Figure 4c; Additional file 12). Taken together, our per nucleotide and per crosslinking site results indicate that high conservation observed for Ts with low CLS values is driven by conserved RNA secondary structures, while Ts with high CLS values are located in exposed, single-stranded RNA regions available for sequence-specific contact with RBPs.

### Large-scale changes of RBP crosslinking site occupancy occur upon nutrient deprivation

**Figure 5:**
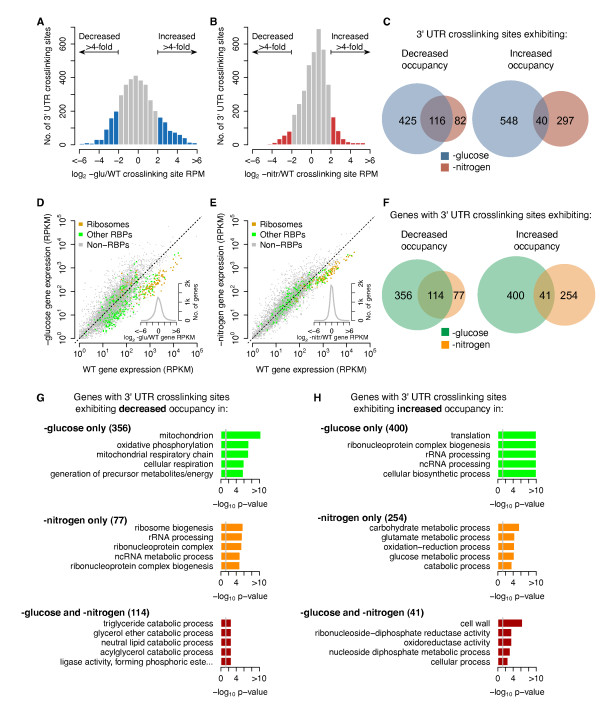
**Nutrient deprivation induces widespread but distinct RBP-crosslinking and mRNA changes**. **(a,b) **Changes in 3' UTR crosslinking site coverage upon glucose (a) or nitrogen (b) starvation. Standard deviations of intra-replicate variation: wild-type (WT), 1.31-fold; glucose starvation, 1.24-fold; nitrogen starvation, 1.15-fold. **(c) **Overlap of 3' UTR crosslinking site changes affected by glucose or nitrogen starvation conditions. **(d,e) **Global changes in mRNA abundance upon glucose (d) or nitrogen (e) starvation. **(f) **Overlap of mRNAs with 3' UTR crosslinking site changes affected by glucose or nitrogen starvation conditions. **(g,h) **Enriched Gene Ontology terms for mRNAs with 3' UTR crosslinking sites with decreased (g) or increased (h) RBP occupancy upon glucose or nitrogen starvation or both. Grey lines indicate *P*-value of 0.05.

To explore RBP-RNA interaction dynamics under changing cellular conditions, we compared gPAR-CLIP read coverage of individual 3' UTR crosslinking sites between glucose or nitrogen starvation and log-phase growth conditions. As we selected non-translating mRNAs for gPAR-CLIP analyses, we cannot distinguish whether the changes in binding site coverage reflect changes in RBP binding or changes in RBP distribution in the sucrose gradient (see Discussion). We only examined crosslinking sites with >5 reads per million mapped reads (RPM) in gPAR-CLIP libraries to ensure confident quantification (Additional file 13; Materials and methods). The intra-replicate variation of crosslinking site read coverage was quantified as standard deviation σ = 1.3-fold (Additional file 13); therefore, we consider crosslinking sites with more than 4-fold (3σ) differences in read coverage between wild type and stress conditions as 'increased' or 'decreased'. We observed >4-fold changes in crosslinking site coverage, also referred to as 'RBP occupancy', for 1,129 of 3,803 (30%) 3' UTR sites upon glucose starvation and for 535 of 3,932 (14%) 3' UTR sites upon nitrogen starvation (1,497 of 3,985 3' UTR sites in either condition, 38%) (Figure 5a,b). Similar distributions of changes were observed for crosslinking sites in 5' UTRs (Additional file 13). Nineteen percent (116 of 623) of crosslinking sites that exhibited decreased RBP occupancy were affected by both conditions, while only 5% (40 of 885) of crosslinking sites that exhibited increased RBP occupancy were affected by both conditions, suggesting that RBP-RNA interaction changes are largely distinct to glucose or nitrogen deprivation (Figure 5c). Similar to the observation that glucose starvation induced more crosslinking site occupancy changes than nitrogen starvation, comparison of mRNA abundance revealed more changes in gene expression upon glucose than nitrogen starvation (Figure 5d,e). Interestingly, mRNA expression of ribosomal subunits and other known RBPs was significantly down-regulated upon glucose (Welch's *t*-test, *P *< 10^-27^) and nitrogen (Welch's *t*-test, *P *< 10^-36^) starvation, suggesting that global suppression of post-transcriptional regulation is a general response to nutrient deprivation.

We next examined the overlap of individual genes with 3' UTR crosslinking sites affected by each stress condition (Figure 5f). Genes harboring 3' UTR crosslinking sites with increased RBP occupancy showed little overlap (41 genes, 6%) between the two conditions; genes harboring crosslinking sites with decreased RBP occupancy showed higher overlap (114 gene, 21%). These data suggest that, for the non-translated mRNA transcriptome, loss of RBP occupancy at crosslinking sites of a larger set of common genes is a general response to nutrient limitation while increased RBP occupancy at crosslinking sites of distinct sets of genes is a nutrient-specific response.

We determined if genes exhibiting common or distinct 3' UTR crosslinking site occupancy changes under nitrogen and glucose starvation conditions had shared biological functions or cytological localization using Gene Ontology (GO) enrichment analysis (Figure 5g,h; Additional file 14). When we analyzed the 356 genes with sites decreased in RBP occupancy only during glucose starvation, mitochondrion-related genes and genes associated with cellular respiration were preferentially affected (Figure 5g, top). Analysis of the 77 genes with sites lost only during nitrogen starvation revealed enrichment for ribosomal components and noncoding RNA processing (Figure 5g, middle). The 114 genes harboring 3' UTR crosslinking sites with decreased coverage under both stress conditions were enriched for fatty acid and lipid catabolism (5g, bottom), consistent with the utilization of stored lipids as energy source in response to nutrient deprivation [45].

Analysis of the 400 genes harboring 3' UTR crosslinking sites with increased occupancy only upon glucose starvation were enriched for terms related to translation (Figure 5g, top). The 254 genes harboring sites with increased RBP occupancy only upon nitrogen starvation were enriched for metabolic processes, including glutamate metabolic processes, which are affected by nitrogen availability (Figure 5h, middle). Of the 41 genes harboring 3' UTR crosslinking sites with increased RBP occupancy under both nitrogen and glucose starvation conditions, 13 (32%) genes represent cellular components of ribosomes or mitochondria (Figure 5h, bottom), consistent with induction of global changes through translational repression and changes in energy metabolism.

In order to determine whether these observations are a result of changes in mRNA abundance, we calculated GO term enrichment of mRNAs up- or down-regulated upon glucose or nitrogen starvation and observed that down-regulated mRNAs are enriched for ribosome- and translation-related genes, while up-regulated mRNAs are enriched for genes related to mitochondrion and metabolic processes (Additional file 15). Therefore, the GO term enrichment of genes with changes in 3' UTR site occupancy cannot be fully explained by GO term enrichment of up- or down-regulated mRNAs. Taken together, these data indicate that general nutrient limitation triggers a remodeling of the post-transcriptional regulatory programs of metabolic pathways, while glucose- and nitrogen-specific stresses affect additional, distinct biological processes.

**Figure 6:**
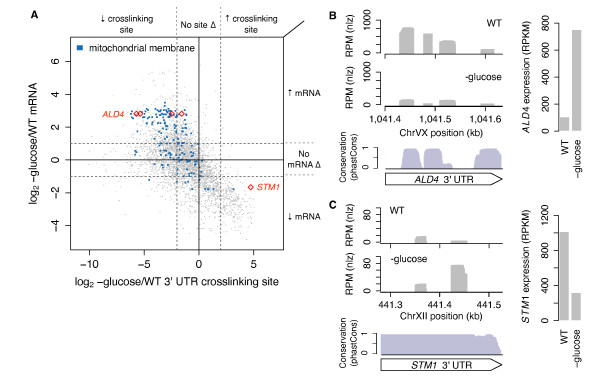
**Glucose starvation induces RBP-crosslinking and mRNA changes associated with mitochondrial processes**. **(a) **Changes in 3' UTR crosslinking site coverage versus changes in the corresponding mRNA upon glucose starvation. Crosslinking sites on genes annotated with the 'mitochondrial membrane' GO term are colored blue. Dotted lines indicate ≥4-fold changes in crosslinking site coverage (vertical) or ≥2-fold change in mRNA expression (horizontal). **(b) ***ALD4 *3' UTR contains four crosslinking sites that decrease two- to eight-fold in RBP occupancy upon glucose starvation and overlap with conserved blocks (red diamonds in (a)). *ALD4 *mRNA expression is up-regulated upon glucose starvation. **(c) ***STM1 *3' UTR contains one crosslinking site that increases in coverage upon glucose starvation (red diamond in (a)). *STM1 *mRNA expression is down-regulated upon glucose starvation. WT, wild type.

We further visualized changes in RBP occupancy of 3' UTR crosslinking sites relative to the changes in corresponding mRNA abundance induced by glucose starvation (Figure 6a; Additional file 16). Since 3' UTR crosslinking sites with decreased RBP occupancy were enriched for mitochondrion-related genes, we examined sites on a subset of these genes encoding mitochondrial membrane components and observed that the crosslinking sites were significantly depleted of RBP occupancy compared to all 3' UTR crosslinking sites (Welch's *t*-test, *P *< 10^-21^), and the mRNAs were significantly up-regulated compared to all genes (Welch's *t*-test, *P *< 10^-28^) (Figure 6a, blue dots). This observation suggests that 3' UTR crosslinking sites on mRNAs encoding mitochondrial membrane components are recognized by repressive RBPs, and that upon glucose deprivation, RBP-binding is attenuated, resulting in increased mRNA levels.

Mitochondrial aldehyde dehydrogenase *ALD4 *mRNA is regulated transcriptionally under stress conditions [46,47]. In our gPAR-CLIP data, the *ALD4 *3' UTR harbors four highly conserved crosslinking sites displaying 2- to 8-fold decreases in RBP occupancy despite a >7-fold increase in *ALD4 *mRNA levels (Figure 6b; Additional file 17). These data suggest that post-transcriptional regulation of *ALD4 *in response to glucose deprivation also occurs through the release of repressive RBP binding at these 3' UTR sites. *STM1*, which encodes a ribosomal subunit-associated protein required for optimal translation under nutrient stress [48], has two 3' UTR crosslinking sites, with one exhibiting >25-fold increased RBP occupancy upon glucose starvation (Figure 6c; Additional file 17). *STM1 *mRNA is conversely down-regulated >3-fold, indicating a potential regulatory role for this site involving mRNA stability and/or decay. Interestingly, *STM1 *mRNA expression is also down-regulated upon nitrogen starvation despite no change in RBP occupancy of this site, pointing to non-overlapping regulatory mechanisms that contribute to *STM1 *regulation in glucose and nitrogen starvation conditions.

**Figure 7:**
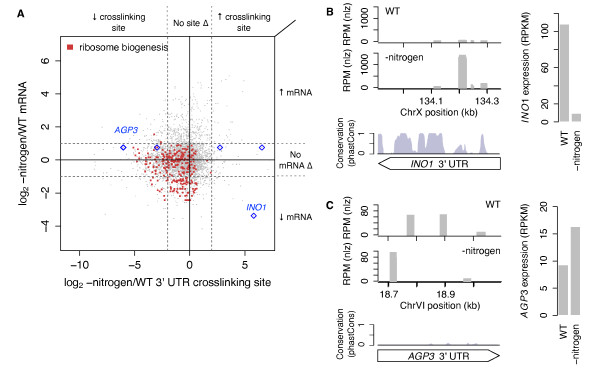
**Nitrogen starvation induces specific RBP-crosslinking and mRNA changes associated with ribosomes and translation-related processes**. **(a) **Global changes in 3' UTR crosslinking site coverage versus changes in the corresponding mRNA upon nitrogen starvation. Crosslinking sites on genes annotated with 'ribosome biogenesis' GO term are colored red. Dotted lines indicate ≥4-fold changes in crosslinking site coverage (vertical) or ≥2-fold change in mRNA expression (horizontal). **(b) ***INO1 *3' UTR contains one crosslinking site that increases in coverage upon nitrogen starvation and falls within a conserved block (blue diamond in (a)). *INO1 *mRNA expression is down-regulated upon nitrogen starvation. **(c) ***AGP3 *3' UTR contains three crosslinking sites that are lost and two crosslinking sites that appear upon nitrogen starvation (blue diamonds in (a)). *AGP3 *mRNA expression is up-regulated upon nitrogen starvation.

Next we explored changes in RBP occupancy of 3' UTR crosslinking sites relative to changes in corresponding mRNA abundance upon nitrogen starvation. 3' UTR crosslinking sites on mRNAs associated with ribosome biogenesis showed significantly greater decrease in RBP occupancy compared to all 3' UTR crosslinking sites (Welch's *t*-test, *P *< 10^-12^) (Figure 7a, red dots; Additional file 18). Inositol-3-phosphate synthase *INO1 *is transcriptionally regulated under stress [49]. *INO1 *3' UTR has four conserved crosslinking sites, one of which exhibits a >50-fold increase in RBP occupancy upon nitrogen starvation despite a >10-fold decrease in *INO1 *mRNA levels (Figure 7b; Additional file 19). These data suggest post-transcriptional regulation of *INO1 *mRNA by a specific RBP-RNA interaction in the 3' UTR. We also identified three crosslinking sites under normal growth conditions in the 3' UTR of *AGP3*, an amino acid permease capable of supplying amino acids as an alternative nitrogen source in nitrogen-poor conditions [50]. RBP occupancy at these sites was completely lost upon nitrogen starvation while two additional sites emerged (Figure 7c; Additional file 19). *AGP3 *mRNA levels moderately increased approximately two-fold (Figure 7c), suggesting complex, combinatorial post-transcriptional regulation of *AGP3 *expression in nitrogen-poor conditions.

## Discussion

RNP complexes exhibit dynamic properties that are sensitive to environmental conditions. For example, granules containing stalled translation pre-initiation complexes are formed under stress but rapidly dissociate when the cell returns to favorable conditions [51]. Despite insight into how particular RNP complexes are affected by stress, global effects of stress on all RBP-RNA interactions have until now remained unexplored. We detect reproducible changes in occupancy for 38% of 3' UTR crosslinking sites on non-translating mRNAs under glucose or nitrogen starvation conditions: loss of RBP occupancy at RBP crosslinking sites was a phenomenon common to both glucose and nitrogen stress conditions, while more distinct sets of crosslinking sites increased RBP occupancy (Figure 5c).

In our current work, we limited our gPAR-CLIP analyses to protein-RNA interactions residing in non-translated RNPs (Figure 1a; Materials and methods), which mediate important functions for mRNA translation, localization, and degradation [52]. Because we have no information on the identities of the RBPs or their distribution in the sucrose gradient, we cannot distinguish whether the changes in RBP coverage represent changes in RBP binding and/or distribution. This is particularly relevant in glucose or nitrogen starvation, as many RBPs redistribute under these conditions [53]. Future comparative gPAR-CLIP analyses on both non-translating RNP and translating RNPs in stress conditions will distinguish changes in RBP binding versus changes in RBP localization.

RNAs are capable of forming complex two- and three-dimensional structures, and some RBPs are known to recognize such structural motifs. For example, She2p mediates the localization of several bud-localized transcripts during cell division by recognizing and binding to specific stem-loop structures in mRNAs [40,41]. Examination of the structural properties of our global RBP crosslinking sites revealed a preference for single-stranded regions, which agrees with previous reports of crosslinking sites of the RNA-binding protein FUS occurring at single-stranded regions directly adjacent to the FUS RNA recognition motif [54]. Unpaired loop and bulge regions can be unstructured or form tertiary structural modules, both of which can be readily recognized by RBPs. In contrast, double-stranded RNAs, in general, do not provide good platforms for RBP binding: structured RNA regions captured by gPAR-CLIP generally had low CLS values (Figure 4), likely resulting from crosslinking and/or RNase T1 cleavage inefficiency. In structured regions, 4-thiouridines are more likely to be locked in U:A or U:G pairing, preventing crosslinking to proteins. In addition, structured regions are less accessible to RNase attack during sequencing fragment preparation, resulting in under-representation in gPAR-CLIP libraries. Nevertheless, despite their low crosslinking efficiencies, Ts in double-stranded, paired RNA regions show extremely high conservation compared to Ts with no crosslinking evidence. These data indicate that RNAs with high secondary structure are evolutionarily conserved and can serve as functional, secondary structure motifs recognized by select RBPs.

RBP binding sites functioning as *cis*-regulatory elements are expected to be under purifying selection. We identified a substantial fraction (35%) of conserved elements in UTRs overlapping RBP crosslinking sites. This represents an underestimation because RBPs and RNAs that are not expressed under our experimental conditions or that fail to crosslink will not be captured. Although crosslinking sites in general are more highly conserved than non-crosslinking sites in UTRs, many sites are not well conserved and might represent species-specific *cis*-regulatory elements that allow adaptation to different environments and stressors.

A preference of RBP binding to 3' UTRs observed in this study and others [13,14] is consistent with the function and evolution of 3' UTRs as major sites for post-transcriptional regulation. Unlike protein-coding regions, 3' UTRs do not directly engage ribosomes during translation and therefore provide accessible platforms for RBP binding and RNP assembly. One important aspect of gene regulation is combinatorial control, which allows a single gene to be controlled by more than one regulator. In our study, 23% of all nucleotides in annotated 3' UTRs were located within RPB crosslinking sites, corresponding to an average of 1 crosslinking site, on average 23 nucleotides long, in every 100 nucleotides. For a median-sized yeast 3' UTR that is 166 nucleotides long [38], there are, on average, 2 RBP crosslinking sites, suggesting that most yeast genes are subject to combinatorial post-transcriptional regulation. Since *S. cerevisiae *lacks post-transcriptional regulation by the highly conserved and pervasive microRNA regulatory pathway, combinatorial regulation by RBPs may play a more prominent role than in organisms with small RNA-mediated post-transcriptional gene regulation.

Unlike focused interrogation of individual RBPs, gPAR-CLIP does not directly identify the RBP that recognizes each crosslinking site. To enable identification of primary sequence motifs recognized by individual RBPs, we searched gPAR-CLIP crosslinking sites located on target mRNAs identified *in vitro *by RIP-Chip for 29 RBPs [12,13] and identified 39 motifs for 15 RBPs (Additional file 20; Materials and methods). Notably, 35 of the sequence motifs derived by gPAR-CLIP differed significantly from previous motif predictions, which were based on scanning whole transcript sequences for enriched k-mers. This discrepancy between primary sequence motifs identified by our gPAR-CLIP data and previous predictions illustrates the potential utility of deriving motifs based on direct *in vivo *evidence of RBP-RNA interactions, which narrows the search space to enhance the signal of *bona fide *primary sequence recognition elements.

## Conclusions

Our study provides a comprehensive map of RBP crosslinking sites across the budding yeast non-translating mRNA transcriptome and for the first time describes the dynamics of mRNA-RBP binding under normal and nutrient-limited growth conditions. Delineating *in vivo *sites of RBP binding will aid in directing future studies for identification of sites responsive to environmental or genetic perturbations, refinement of primary sequence and secondary structural elements recognized by specific RBPs, and elucidation of the complex network of regulatory processes that contribute to regulation of expression of each individual mRNA. gPAR-CLIP is readily applicable to other organisms for profiling global RNA-protein interactions underlying post-transcriptional regulation and the effects of environmental perturbations upon these interactions.

## Materials and methods

### Strains, media and growth conditions

The following strains were used in this study: wild-type BY4742 (*MAT*α *his3*Δ1 *leu2*Δ0 *lys2*Δ0 *ura3*Δ0), TAP-tagged strains picked from TAP-tagged yeast strain collection (*MAT***a ***his3*Δ*1 leu2*Δ*0 met15*Δ*0 ura3*Δ*0 YFG-TAP::HIS5) *[55]. Strains were grown at 30°C with vigorous shaking (250 rpm) in synthetic defined media, supplemented with 200 µM 4sU (Sigma-Aldrich 440736; St. Louis, MO, USA, to OD_600 _= 0.7 to 0.8. Starvation was performed by pelleting cells for 5 minutes at 3,000Xg at room temperature, discarding all media, rinsing once with H_2_O, and resuspending cells in an equal volume of synthetic defined media without glucose or nitrogen (supplemented with 200 µM 4sU). Cells were returned to 30°C with shaking for 2 h. Strains used are defective in uracil synthesis (*ura3*Δ) and readily take up 4sU from the media. Inside the cell, 4sU is converted by Fur1p (uracil phosphoribosyltransferase) to 4-thiouridine monophosphate that can be incorporated during RNA synthesis.

### Estimation of 4sU incorporation rates

4sU incorporation rates were measured as described [56]. Briefly, RNA samples isolated from cells grown in the presence or absence of 4sU were dissolved in 100 µl of 12 mM Tris buffer, pH 7, and their A_260 _absorption was adjusted to the same value. A_330 _was measured for both samples using a Q6 quartz cuvette with 1 mm light path in a Thermo Scientific BioMate 3 UV-Vis spectrophotometer. 4sU incorporation rates per kilobase RNA were calculated as 500 × [(A_330_(+4sU)) - (A_330_(-4sU))]/A_260_. 4sU was incorporated at roughly four 4sU per kilobase of transcript, with little interference with cell growth and only minor changes in gene expression (Additional file 3).

### gPAR-CLIP procedures

#### UV crosslinking

Mid-log phase cultures (50 ml; OD_600 _of 0.7 to 0.8) were pelleted for 5 minutes at 3,000Xg at room temperature, resuspended in 2 ml of 1× HBSS (Invitrogen 14025; Grand Island, NY, USA) and transferred to a 60 mm cell culture dish (BD Biosciences 353002; San Jose, CA, USA), placed on ice, and irradiated with 365 nm UV at 150 mJ/cm^2 ^four times using a UVP CL-1000L UV crosslinker. The cells were then pelleted for 2 minutes at 5,000Xg at 4°C. After removing 1× HBSS, the cells were frozen in liquid nitrogen.

#### Extract preparation

Crosslinked cells were resuspended in polysome lysis buffer (20 mM HEPES pH 7.5, 140 mM KCl, 1.5 mM MgCl_2_, 1% Triton X-100, 1× Complete Mini Protease Inhibitor EDTA-free (Roche Applied Science 1 836 170; Indianapolis, IN, USA), 0.2 U/µl SUPERase In (Invitrogen AM2696)), mixed with half a volume of acid-washed glass beads, and lysed by vortexing four times at 4°C, 1 minute each with a 1 minute incubation on ice in between. Cell debris was removed by centrifugation for 5 minutes at 1,300Xg at 4°C. The supernatant was cleared by 20,000Xg spin for 10 minutes at 4°C.

#### Ribosome depletion using sucrose density gradients

Sucrose density gradients (15 to 50% (w/v)) were prepared in Beckman polycarbonate centrifugation tubes (11 × 34 mm) by sequentially layering and freezing 0.24 ml of 50%, 41.25%, 32.5%, 23.75% and 15% sucrose dissolved in polysome gradient buffer (20 mM HEPES pH 7.5, 140 mM KCl, 5 mM MgCl_2_). Gradients were thawed overnight at 4°C before use. 100 µl of clarified lysate was loaded on top of a gradient, centrifuged for 1 h at 54,000 rpm at 4°C using a TLS-55 rotor in an Optima MAX-E ultracentrifuge (Beckman Coulter; Palo Alto, CA, USA). The top 600 µl of the gradient were recovered and supplemented with 2 µl of SUPERase In (20 U/µl).

#### Chemical biotinylation and polyA selection

Sixty microliters of freshly prepared 10 mM EZ-Link NHS-SS-Biotin (Pierce 21441; Rockford, IL, USA) dissolved in dimethylformamide was added to the recovered lysate and incubated on a Nutator for 2 h at 4°C; 50 µl of 5 M NaCl was added to increase the total salt concentration to 0.5 M. Biotinylated lysate was mixed with 1 mg of oligo(dT)_25 _magnetic beads (NEB S1419S; Ipswich, MA, USA), then incubated on a Nutator for 30 minutes at 4°C. The beads were washed four times with ice-cold hybridization buffer (10 mM HEPES pH 7.5, 0.5 M NaCl, 1 mM EDTA) and the RNAs were eluted by incubating beads with 500 µl of elution buffer (10 mM HEPES pH 7.5, 1 mM EDTA) and heating at 65°C for 3 minutes. The eluted sample was transferred to a new tube and mixed with 55 µl of 10× phosphate-buffered saline (PBS).

#### Streptavidin binding and RNase T1 digestion

PolyA-selected samples were mixed with 1 mg of streptavidin M280 Dynabeads (Invitrogen 112-05D) and incubated on a Nutator for 30 minutes at 4°C. The beads were washed three times with 1× PBS, then incubated with 20 µl of 50 U/µl RNase T1 (Fermentas EN0541; Waltham, MA, USA, 1:20 dilution in 1× PBS) at 22°C for 15 minutes on an Eppendorf Thermomixer (15 s shaking at 1,000 rpm followed by a 2 minute rest interval), followed by a 5 minute incubation on ice. Beads were washed twice with wash buffer (1× PBS, 0.1% SDS, 0.5% deoxycholate, 0.5% NP-40), twice with high-salt wash buffer (5× PBS, 0.1% SDS, 0.5% deoxycholate, 0.5% NP-40) and twice with 1× PNK buffer (50 mM Tris pH 7.4,10 mM MgCl_2_, 0.5% NP-40).

#### On-bead CIP treatment

Beads were incubated with 20 µl of CIP mix (50 mM Tris pH 7.9, 100 mM NaCl, 10 mM MgCl_2_, 0.5 U/µl calf intestinal alkaline phosphatase (CIP); NEB M0290S) at 37°C for 15 minutes, with 15 s shaking at 1,000 rpm followed by a 2 minute rest interval on a Thermomixer. After CIP treatment, beads were washed twice with 1× PNK+EGTA buffer (50 mM Tris pH 7.4, 20 mM EGTA, 0.5% NP-40) and twice with 1× PNK buffer.

#### On-bead 3' DNA linker ligation

Beads were incubated with 20 µl of ligation mix (50 mM Tris pH 7.4, 10 mM MgCl_2_, 0.5 mM DTT, 2 µM pre-adenylated 3' DNA linker, 25% PEG-8000, 10 U/µl T4 RNA ligase 2, truncated K227Q; NEB M0351S) at 16°C overnight (≥16 h), with 15 s shaking at 1,000 rpm followed by a 2 minute interval on a Thermomixer. After linker ligation, beads were washed three times with 1× PNK+EGTA buffer.

#### SDS-PAGE and nitrocellulose transfer

Beads were mixed with 12 µl of 1× PNK+EGTA buffer, 3 µl of freshly made 1 M DTT and 15 µl of 4× NuPAGE LDS sample buffer (Invitrogen NP0007), and incubated at 70°C for 10 minutes. Beads were removed, and the supernatant was loaded onto NuPAGE 4-12% Bis-Tris gel (Invitrogen NP0335BOX) and run at 150 V for 35 minutes. The gel was transferred to Protran BA 85 nitrocellulose membrane (pore size 0.45 µm, Whatman 10402594; St. Louis, MO, USA) using Novex wet transfer at 30 V for 1 h. A broad band from 31 kDa up to the top of the gel was excised, cut into small pieces, and transfered into a microfuge tube.

#### RNA isolation and purification

Excised membranes were incubated with 500 µl of 4 mg/ml Proteinase K prepared in 1× PK buffer (100 mM Tris pH 7.5, 50 mM NaCl, 10 mM EDTA) for 20 minutes at 37°C on a Thermomixer. We added 500 µl of 7 M urea prepared in 1× PK buffer to the tube followed by another 20 minute incubation at 37°C. The Proteinase K digestion reaction was mixed with 1 ml of phenol:chloroform:isoamyl alcohol 25:24:1 (Sigma-Aldrich P2069) by vortexing and spun for 5 minutes at 20,000Xg. The liquid phase was transferred into a new tube, mixed with 125 µl of 3 M NaOAc, 2.5 ml of 100% ethanol and 1 µl of 15 mg/ml glycoblue (Invitrogen AM9516), and precipitated for 2 h at -80°C. RNAs were collected by centrifugation for 20 minutes at 20,000Xg at room temperature followed by two washes with cold 75% ethanol.

#### RNA 5' end phosphorylation

RNA pellets were air-dried briefly, resuspended in 10 µl of PNK mix (70 mM Tris pH 7.6, 10 mM MgCl_2_, 5 mM DTT, 1 mM ATP, 1 U/µl T4 polynucleotide kinase (NEB M0201S), 1 U/µl SUPERase In) and incubated at 37°C for 30 minutes. The reaction was combined with 90 µl of H_2_O and 100 µl of phenol:chloroform:isoamyl alcohol 25:24:1, mixed well and spun for 5 minutes at 20,000Xg. The liquid phase was mixed with 12.5 µl of 3 M NaOAc, 250 µl of 100% ethanol, 1 µl of 15 mg/ml glycoblue and precipitated for 2 h at -80°C. RNAs were collected by centrifugation for 20 minutes at 20,000Xg at room temperature, followed by two washes with cold 75% ethanol.

#### 5' RNA linker ligation

RNA pellets were resuspended in 10 µl of ligation mix (50 mM Tris pH 7.5, 10 mM MgCl_2_, 10 mM DTT, 1 mM ATP, 0.1 mg/ml bovine serum albumin, 2 µM 5' RNA linker, 1 U/µl T4 RNA ligase (Fermentas EL0021), 1 U/µl SUPERase·In, 10% DMSO) and incubated at 15°C for 2 h.

#### RNA size selection

Ligation reaction was terminated by adding 10 µl of 2× formamide gel loading buffer (Invitrogen AM8546G), heated for 2 minutes at 70°C and then quickly chilled on ice. Samples were loaded onto a 6% TBE UREA gel (Invitrogen EC6865BOX) and run at 150 V for 45 minutes. After staining with 1× Sybr Gold Stain (Invitrogen S-11494), a gel piece corresponding to a 70 to 90 nucleotide RNA (80 to 100 nucleotide single-stranded DNA) was excised, crushed, and soaked in 400 µl of 0.3 M NaOAc overnight at room temperature. After removing gel pieces, the solution was combined with 1 ml of 100% ethanol and 1 µl of 15 mg/ml glycoblue and precipitated for 2 h at -80°C. RNAs were collected by centrifugation for 20 minutes at 20,000Xg at room temperature, followed by two washes with cold 75% ethanol. After brief drying, RNAs were resuspended in 15 µl of H_2_O.

#### RT-PCR

The ligated RNA (10 μl) was combined with 2 μl of 5 µM RT primer, heated at 65°C for 5 minutes, and then quickly chilled on ice, and followed by the addition of 1 µl of 10 mM dNTP, 1 μl of 0.1 M DTT, 4 μl of 5× First strand buffer, 1 μl of SUPERase·In (20 U/µl) and 1 µl of SuperScript III Reverse transcriptase (Invitrogen 18080-093, 200 U/µl). The RT reaction was kept at 50°C for 45 minutes, 55°C for 15 minutes and 90°C for 5 minutes. A test PCR was performed with 2.5 µl of RT product in 50 µl PCR mix: 1× AccuPrime PCR buffer I, 0.5 µM P5 long primer, 0.5 µM P7 primer, 0.2 µl AccuPrime Taq High Fidelity (Invitrogen 12346-086, 5 U/µl). PCR was carried out with an initial 3 minute denaturation at 98°C, followed by 14 to 22 cycles of 80 s denaturation at 98°C, 90 s annealing and extension at 65°C, and termination with a final 5 minute extension at 65°C. PCR product (15 µl) was collected after 14, 18, and 22 cycles and analyzed on a 10% TBE gel (Invitrogen EC6275BOX) at 150 V for 1 h to determine the optimal amplification cycles (the lowest cycle number required to generate 96 to 116 bp amplicons detected by Sybr Gold staining).

#### Preparation of sequencing libraries

A 50 µl PCR reaction was carried out with the determined cycle number. Amplicons were purified using DNA clean and concentrator-5 (Zymo D4013; Irvine, CA, USA), run on 10% TBE gels at 150 V for 1 h and stained with Sybr Gold. A gel piece corresponding to 96 to 116 bp DNA was excised, crushed, and soaked overnight in 400 µl 0.3 M NaOAc at room temperature. After removing gel pieces, the solution was combined with 1 ml of 100% ethanol and 1 µl of 15 mg/ml glycoblue and precipitated for 2 h at -80°C. DNAs were collected by centrifugation for 20 minutes at 20,000Xg at room temperature, followed by two washes with cold 75% ethanol. After brief drying, amplicons were resuspended in 20 µl of H_2_O. Purified amplicons (5 µl) were used to seed a second round of PCR in 50 µl: 1× AccuPrime PCR buffer I, 0.5 µM Illumina Primer A, 0.5 µM Illumina Primer B, 0.2 µl AccuPirme Taq High Fidelity for 6 to 12 cycles. Second PCR amplicons were purified with DNA clean and concentrator-5 (Zymo D4013) and sequenced on an Illumina HiSeq 2000 sequencer.

### Puf3p PAR-CLIP procedures

Puf3p PAR-CLIP was performed similarly to gPAR-CLIP with the following modifications. The *PUF3-TAP::HIS5 *strain was cultured and UV-crosslinked as in gPAR-CLIP. Cells were lysed in 1× PBS, 0.5% NP-40, 1× Complete Mini Protease Inhibitor, EDTA-free and cleared by sequential spins at 1,300Xg for 5 minutes and 20,000Xg for 10 minutes at 4°C. The clarified lysate was passed through a Costar Spin-X filter (Corning CLS8160; St. Louis, MO, USA), mixed with RNase T1 (Fermentas EN0541) to 1 U/µl, and incubated at 22°C for 15 minutes followed by a 5 minute incubation on ice. The lysate was then directly mixed with IgG magnetic beads (prepared by coupling rabbit IgG (Sigma-Aldrich I5006) to Dynabeads M-270 Expoxy (Invitrogen 143-01)) to pull down Puf3p::TAP. RNase T1 digestion, CIP treatment, and 3' DNA linker ligation were performed as described in gPAR-CLIP. Afterwards, 5' end phosphorylation was performed on-bead in 20 µl of PNK mix (70 mM Tris pH 7.6, 10 mM MgCl_2_, 5 mM DT, 1 µl P32 rATP (6000 Ci/mmol 10 mCi/ml Perkin Elmer BLU502Z500UC; Waltham, MA, USA), 1 U/µl T4 polynucleotide kinase) and incubated at 37°C for 15 minutes. ATP (2 µl of 10 mM) was added to the mix and the reaction was incubated for 10 minutes. After SDS-PAGE and transfer, crosslinked RNAs were visualized by autoradiography and the corresponding Puf3p band was excised. The remaining steps were carried out as described in gPAR-CLIP procedures, omitting the 5' end phosphorylation step.

### mRNA-seq procedures

Yeast strains were grown under normal and starvation conditions described above in the presence of 4sU. Additional replicate mRNA-seq libraries were prepared with yeast strains grown under normal conditions without the addition of 4sU.

Total RNAs were extracted with acid-phenol:chloroform, pH 4.5 with isoamyl alcohol, 25:24:1 (Ambion; Grand Island, NY, USA). Replicate, strand-specific total mRNA-seq libraries were prepared in parallel using the two linker ligation protocol as described [57].

For preparation of ribo- mRNA-seq libraries, extract preparation, and ribosome depletion using sucrose density gradients were carried out as described in the gPAR-CLIP procedure (avoiding UV crosslinking). PolyA+ mRNAs were enriched using oligo(dT)25 beads and converted into sequencing libraries as described [57].

### Oligos for constructing gPAR-CLIP, PAR-CLIP, and mRNA-seq libraries

Oligos used in this study were synthesized by Integrated DNA Technologies, except the 5' RNA linker, which was synthesized by Dharmacon (Waltham, MA, USA).

#### Barcodes

3' DNA linker oligos (5' phosphorylated, and 3' block with inverted deoxythymindine):

Index 1: 5' pATCACGTCGTATGCCGTCTTCTGCTTGidT 3'

Index 2: 5' pCGATGTTCGTATGCCGTCTTCTGCTTGidT 3'

Index 3: 5' pTTAGGCTCGTATGCCGTCTTCTGCTTGidT 3'

Index 4: 5' pTGACCATCGTATGCCGTCTTCTGCTTGidT 3'

Index 5: 5' pACAGTGTCGTATGCCGTCTTCTGCTTGidT 3'

Index 6: 5' pGCCAATTCGTATGCCGTCTTCTGCTTGidT 3'

Index 7: 5' pCAGATCTCGTATGCCGTCTTCTGCTTGidT 3'

Index 8: 5' pACTTGATCGTATGCCGTCTTCTGCTTGidT 3'

Pre-adenylation of 3' DNA linker oligos was performed with Mth RNA ligase (5' DNA adenylation kit, NEB E2610S) following the vendor's instructions.

#### 5' RNA linker

5' GUUCAGAGUUCUACAGUCCGACGAUC 3'.

#### Barcoded RT primers

Index 1: 5' CAAGCAGAAGACGGCATACGACGTGAT 3'

Index 2: 5' CAAGCAGAAGACGGCATACGAACATCG 3'

Index 3: 5' CAAGCAGAAGACGGCATACGAGCCTAA 3'

Index 4: 5' CAAGCAGAAGACGGCATACGATGGTCA 3'

Index 5: 5' CAAGCAGAAGACGGCATACGACACTGT 3'

Index 6: 5' CAAGCAGAAGACGGCATACGAATTGGC 3'

Index 7: 5' CAAGCAGAAGACGGCATACGAGATCTG 3'

Index 8: 5' CAAGCAGAAGACGGCATACGATCAAGT 3'

#### P7 primer

5' CAAGCAGAAGACGGCATACGA 3'.

#### P5 long primer

5' AATGATACGGCGACCACCGACAGGTTCAGAGTTCTACAGTCCGA 3'.

#### Illumina primer A

5' AATGATACGGCGACCACCGA 3'.

#### Illumina primer B

5' CAAGCAGAAGACGGCATACGA 3'.

### Data processing of Illumina HiSeq sequencing reads

gPAR-CLIP, PAR-CLIP, and mRNA-seq reads were processed to remove linkers, sorted into libraries based on six-nucleotide barcodes (underlined residues above), and removed if they were low quality (Additional file 2). First, reads with a perfect match to a barcode were successfully sorted, followed by reads with one mismatch to a barcode. If both barcodes were perfectly matched in a read or both barcodes were found with one mismatch, the 3'-most barcode was chosen; 99.97% of reads were successfully sorted into libraries under these rules. Next, reads were removed if they met any of the following criteria: <18 nucleotides, only homopolymer As, missing 3' adapter, 5'-3' adapter ligation products, 5'-5' adapter ligation products, low quality (more than 4 bases with quality scores below 10 or more than 6 bases with a quality score below 13); 97.0% of gPAR-CLIP, 80.0% of PAR-CLIP, and 99.7% of mRNA-seq reads passed these filters. High quality reads were mapped to the *S. cerevisiae *genome version S288C with Bowtie [58] using the following parameters: -v 3 (map with up to 3 mismatches), -k 275 (map at up to 275 loci), --best, and --strata. Mapped reads were annotated using custom scripts to known genomic elements in the S288C genome (sacCer3, April 2011) including external UTR annotations [59,60]. Additional file 1 provides read counts at each processing step.

### Assessing data reproducibility

To determine mRNA-seq and gPAR-CLIP replicate library reproducibility, we calculated replicate correlation using normalized read counts (RPM) of each gene. Pearson correlation coefficients for mRNA-seq libraries ranged from 0.984 to 0.994, while coefficients for gPAR-CLIP libraries ranged from 0.967 to 0.971. Due to high reproducibility, subsequent measures of read coverage represent averages of two biological replicate libraries. To determine if the addition of 4sU to growth media substantially alters transcription, biological replicate mRNA-seq libraries were generated from wild-type yeast grown under normal conditions without the addition of 4sU. These replicate libraries had a Pearson correlation coefficient of 0.988, indicating high reproducibility, and a Pearson correlation coefficient of 0.982 when compared to wild-type yeast grown in the presence of 4sU.

### Calculation of per-nucleotide crosslinking scores

To measure RBP crosslinking strength, we calculated a crosslinking score for each genomic T position as the RPM coverage from reads with a T-to-C at that position. Because transcript abundance varies greatly, from zero to tens of thousands of copies, T-to-C coverage of crosslinking sites on highly expressed genes would be preferentially higher than T-to-C coverage of crosslinking sites of lowly expressed genes. To avoid this bias, we normalized T-to-C RPMs to length-normalized transcript abundances (RPKM) from our mRNA-seq libraries. Two percent of Ts with T-to-C RPM coverage in gPAR-CLIP libraries were located on genes that lacked mRNA-seq coverage and were thus removed from further analysis. To adjust for the additional kilobase normalization factor used in RPKM, ratios of gPAR-CLIP RPM:mRNA-seq RPKM were multiplied by a factor of 1,000.

### Calculation of RBP crosslinking sites

#### Generation of read clusters from gPAR-CLIP libraries

All six gPAR-CLIP libraries were aggregated into one large dataset to generate read clusters. A read cluster was defined as a continuous stretch of nucleotides covered by at least one gPAR-CLIP read harboring one or two T-to-C conversion events. This step resulted in 84,136 gPAR-CLIP clusters and 1,915 Puf3p PAR-CLIP clusters.

#### Defining crosslinking site boundaries

Manual inspection of read clusters revealed long (>100 nucleotide) regions covered by gPAR-CLIP reads containing one or more distinct peaks indicative of distinct crosslinking sites. To distinguish between read peaks within long read clusters and trim low read coverage surrounding strong single peaks, we fit a Gaussian smoothed curve (normal kernel function, bandwidth 21) to each read cluster and used the inflection points of this curve to define the boundaries of individual crosslinking sites. This step resulted in 91,290 gPAR-CLIP crosslinking sites and 1,915 Puf3p PAR-CLIP crosslinking sites.

#### Calculating read coverage of crosslinking sites

From the set of RBP crosslinking sites derived from all gPAR-CLIP libraries, we determined read coverage for each site from each individual library by calculating the average RPM covering each nucleotide in the crosslinking site. This coverage was divided by the RPKM of the associated gene and multiplied by 1,000 to enable direct comparison of RBP occupancy of crosslinking sites between growth conditions.

#### Assigning FDR to each crosslinking site

A small fraction of T-to-C mismatches in gPAR-CLIP reads likely represent sequencing error instead of crosslinking events, so crosslinking sites derived from this error were removed. We repeated the crosslinking site generation steps using mRNA-seq reads with one or two T-to-C mismatches, which represent the rate of T-to-C sequencing error for the Illumina HiSeq platform. For each gPAR-CLIP and mRNA-seq crosslinking site, we calculated the T-to-C conversion rate as the number of reads with T-to-C conversion events divided by the number of total reads covering Ts. gPAR-CLIP and mRNA-seq crosslinking sites were binned into groups based on total read coverage. For each gPAR-CLIP crosslinking site in each bin, we determined the proportion of mRNA-seq crosslinking sites with a higher T-to-C conversion rate than the gPAR-CLIP crosslinking site. This proportion represents the FDR for that gPAR-CLIP crosslinking site. Using a strict 1% FDR threshold, we identify 80,883 gPAR-CLIP crosslinking sites.

### Effect of counting statistics on error in crosslinking site coverage measurement

Read coverage of replicate gPAR-CLIP crosslinking sites was analyzed to measure reproducibility. For each site, we compared the number of reads coming from one replicate library to the total number of reads from both libraries. Perfect reproducibility would result in a ratio of 1:2. We binned crosslinking sites based on total RPM and calculated the standard deviation of these ratios for each bin. We predicted the standard deviation for counting statistics by binomial partitioning of total reads for each crosslinking site in each bin between the two replicates. When the total number of reads was below 5 RPM, binomial partitioning predominantly contributed to replicate variation (Additional file 13). Above 5 RPM, replicate variation stabilized, and counting statistics error contributed little to replicate error.

### Conservation analysis

phastCons conservation scores for each genomic nucleotide were downloaded from Siepel *et al*. [39]. Ts with CLSs were grouped into 5' UTR, CDS, and 3' UTR regions and then ranked and binned by CLS so each bin overlapped adjacent bins by 50%. phastCons scores in each bin were averaged. As controls, Ts with no CLS were grouped in 5' UTR, CDS, and 3' UTR regions, randomly ranked, and binned as described. phastCons scores in each bin were averaged. Controls were calculated ten times for each region.

### Unpaired probability analysis

The unpaired probability of each genomic position was calculated using RNAplfold [42] from the ViennaRNA package version 1.8.5 using a span of 40 nucleotides and an averaging window of 80 nucleotides. Ts with CLSs were grouped into 5' UTR, CDS, and 3' UTR regions and then ranked and binned by CLS so each bin overlapped adjacent bins by 50%. Unpaired probabilities in each bin were averaged. As controls, Ts with no CLS were grouped into 5' UTR, CDS, and 3' UTR regions, randomly ranked, and binned. The unpaired probabilities in each bin were averaged.

### Crosslinking site pairedness analysis

Genomic regions corresponding to crosslinking sites were extended to 80 nucleotides centered on the original crosslinking site. These sequences were subjected to folding using RNAfold [61] from the ViennaRNA package version 1.8.5, and the minimum free energy structures were extracted. Predicted structures were aligned and ranked by average crosslinking site CLS and divided into 100 equally sized, non-overlapping bins. The percentage of nucleotides predicted to be unpaired at each position in each bin was computed. Selected structures from low, middle, and high CLS bins were visualized using VARNA [62].

### Enriched motif analysis

gPAR-CLIP crosslinking sites passing a 5% FDR threshold from genes identified as RBP targets by RIP-Chip experiments [12,13] were analyzed by MEME [63]. 5' UTR, CDS, and 3' UTR crosslinking sites were analyzed separately, and third-order Markov models based on all 5' UTR, CDS, or 3' UTR regions were used to model background nucleotide compositions. Because gPAR-CLIP crosslinking sites on each target might represent a combination of RBP recognition sites, we implemented MEME using the -mods zoops parameter to allow zero or one motif to be found in each site. The following parameters were also used: -evt 20, -minw 6, and -maxw 15.

### Gene Ontology enrichment analysis

GO analysis was performed on genes harboring 3' UTR crosslinking sites that were four-fold up- or down-regulated upon glucose or nitrogen starvation or both. The topGO R Bioconductor package was implemented using Fisher's exact test for enrichment and Bonferroni correction of *P*-values to adjust for multiple testing [64]. Up to 20 GO terms were reported with a *P*-value <0.01.

### Data availability

Raw sequence data are available through the NCBI's Gene Expression Omnibus [65] using series entry GSE43747.

## Abbreviations

4sU: 4-thiouracil; BP: branch point; CDS: coding sequence; CIP: calf intestinal alkaline phosphatase; CLIP: UV crosslinking followed by immunopurification; CLS: crosslinking score; DTT: dithiothreitol; FDR: false discovery rate; GO: Gene Ontology; gPAR-CLIP: global PAR-CLIP; PAR-CLIP: global photoactivatable-ribonucleoside-enhanced crosslinking and immunopurification; PBS: phosphate-buffered saline; RBP: RNA-binding protein; RIP-Chip: immunoprecipitation of RNA-binding protein and microarray; RNP: ribonucleoprotein; RPKM: reads per million mapped reads per kilobase; RPM: reads per million mapped reads; SELEX: systematic evolution of ligands by exponential enrichment; UTR: untranslated region.

## Competing interests

The authors declare that they have no competing interests.

## Authors' contributions

MAF, TH, and JKK conceived the project; TH and JKK developed the protocols and designed the experiment; TH carried out all of the experiments. MAF conducted the majority of bioinformatics analysis with advice from TH and JKK; AK, YY, and ZJL contributed to RNA structure analysis. MAF, TH, and JKK prepared the figures and wrote the manuscript. All authors read and approved the final manuscript.

## Supplementary Material

Additional file 1 **Sequencing and mapping statistics**. Read counts and T-to-C conversion rates for all gPAR-CLIP, mRNA-seq, and PAR-CLIP libraries. Click here for file

Additional file 2 **Pipeline for generating crosslinking scores and crosslinking sites**. Processing steps used to generate crosslinking scores and crosslinking sites from gPAR-CLIP and mRNA-seq data. Click here for file

Additional file 3 Computational identification of crosslinking sites. **(A) **Illustration of sequence block generation, Gaussian distribution fitting, and cluster segmentation to identify individual crosslinking sites. **(B) **Pearson correlation coefficients for all gPAR-CLIP and mRNA-seq replicate libraries based on gene RPM values. **(C) **Separation of T-to-C sequencing errors from crosslinking-induced mismatches. Plotted for each cluster is T-to-C RPM coverage versus total RPM coverage from gPAR-CLIP or mRNA-seq libraries. **(D) **Percentage of annotated 5' UTR, CDS, and 3' UTR regions with at least one crosslinking site with >5 RPM. Click here for file

Additional file 4 **Table of gPAR-CLIP crosslinking sites**. Data include crosslinking site coverage in gPAR-CLIP libraries, FDRs, conservation scores, and unpaired probabilities. Click here for file

Additional file 5 Visualization of crosslinking site periodicity. Distribution of start-to-start nucleotide distances between 5' UTR, CDS, and 3' UTR read clusters from gPAR-CLIP and mRNA-seq libraries. Only distances from gPAR-CLIP CDS read clusters were enriched for multiples of 3 (red dots). Click here for file

Additional file 6 **Table of gene expression in mRNA-seq libraries**. Values represent reads per million mapped reads per kilobase of transcript (RPKM). Click here for file

Additional file 7 Table of Puf3p PAR-CLIP crosslinking sites. Data include Puf3p recognition motif and whether the crosslinking site was identified in gPAR-CLIP libraries. Click here for file

Additional file 8 Analysis and comparison of PAR-CLIP-identified Puf3p targets. **(A) **Puf3p PAR-CLIP identified crosslinking sites in 147 (67%) of the 220 Puf3p target mRNA identified by RIP-Chip; 174 Puf3p RIP-Chip-identified target mRNAs contain the Puf3p recognition motif UGUAAAUA [12,13]. Puf3p PAR-CLIP identified motif-containing crosslinking sites in 76 (44%) of these mRNAs and in 265 additional mRNAs, suggesting post-transcriptional regulation by Puf3p for these 265 novel targets. **(B) **GO enrichment analysis of 265 PAR-CLIP-identified, motif-containing Puf3p targets. Results are consistent with Puf3p's role in localization, deadenylation, and repression of mRNAs encoding proteins destined for the mitochondria [29]. **(C) **Individual replicate coverage of *COX17 *3' UTR in gPAR-CLIP with average coverage as shown in Figure 2b. Click here for file

Additional file 9 Table of putative Puf3p targets. Data include identification of the Puf3p recognition motif. Click here for file

Additional file 10 **Table of crosslinking scores in all gPAR-CLIP libraries**. Click here for file

Additional file 11 Analysis of crosslinking scores and conservation of genomic Ts in starvation conditions. **(A) **Cumulative distribution of CLSs from 5' UTR, CDS, and 3' UTR regions. **(B) **Individual replicate coverage of ATG8 and TOM40 3' UTRs in gPAR-CLIP with average coverage as shown in Figure 3d. **(C) **Mean phastCons scores for Ts ranked and binned by CLSs. Control lines represent mean phastCons scores of randomly ranked and binned Ts with no CLS, repeated ten times. Click here for file

Additional file 12 Analysis of RNA secondary structure in starvation conditions. **(A) **Mean unpaired probability scores for Ts ranked and binned by CLSs. Control lines represent mean unpaired probability of randomly ranked and binned Ts with no CLS, repeated ten times. **(B) **Heatmaps of pairedness of 5' UTR and CDS crosslinking sites ranked by average crosslinking site CLS. **(C) **Percentage of Ts ranked and binned by CLSs in conserved secondary structural elements as defined by RNAz [43,44]. Control lines represent percentage of randomly ranked and binned Ts with no CLS in conserved secondary structural elements, repeated ten times. Click here for file

Additional file 13 Intra-replicate variation of crosslinking site coverage and global changes in 5' UTR crosslinking sites. **(A) **Determination of minimum crosslinking site coverage required for comparison of sites across environmental conditions. We chose 5 RPM as the minimum crosslinking site coverage needed for confident quantification since, at this coverage, the standard deviation of the fraction of crosslinking site reads coming from one replicate library stabilized at <0.2. Shown are data from wild-type (WT) replicate libraries; similar results were obtained for all library types. **(B) **Intra-replicate variation of 3' UTR crosslinking sites in WT and glucose (left) or nitrogen (right) starvation conditions. Dotted lines represent three standard deviations from the mean and correspond to approximately four-fold change between WT replicates. **(C) **Same as (A) but for 5' UTR crosslinking sites. **(D) **Global changes in 5' UTR crosslinking site coverage upon glucose (left) or nitrogen (right) starvation. Click here for file

Additional file 14 Table of enriched Gene Ontology terms. Table includes data for genes with more than four-fold increased and/or decreased 3' UTR crosslinking sites in nitrogen and/or glucose starvation conditions. Only GO terms with *P*-value <0.001 are reported. Click here for file

Additional file 15 Assessment of crosslinking site and mRNA changes in starvation conditions. **(A,B) **Enriched GO terms of genes up- and down-regulated upon glucose (A) or nitrogen (B) starvation. **(C) **The number and percentage of 3' UTR crosslinking sites with indicated changes in crosslinking site coverage and corresponding mRNA expression upon glucose starvation. **(D) **The number and percentage of 3' UTR crosslinking sites with indicated changes in crosslinking site coverage and corresponding mRNA expression upon nitrogen starvation. Click here for file

Additional file 16 Global changes in 3' UTR crosslinking site upon glucose starvation. Changes in crosslinking site coverage from one replicate library each of wild-type (WT) and glucose starvation conditions are plotted versus changes in the corresponding mRNA from one replicate library each of WT and glucose starvation conditions. Dotted lines and colors are as in Figure 6a. Click here for file

Additional file 17 Changes in 3' UTR crosslinking sites on *ALD4 *and *STM1 *upon glucose starvation. Same as Figure 6b,c but showing crosslinking site coverage and mRNA expression in individual replicate libraries. Click here for file

Additional file 18 Global changes in 3' UTR crosslinking site upon nitrogen starvation. Changes in crosslinking site coverage from one replicate library each of wild-type (WT) and nitrogen starvation conditions are plotted versus changes in the corresponding mRNA from one replicate library each of WT and nitrogen starvation conditions. Dotted lines and colors are as in Figure 7a. Click here for file

Additional file 19 Changes in 3' UTR crosslinking sites on *INO1 *and *AGP3 *upon glucose starvation. Same as Figure 7b,c but showing crosslinking site coverage and mRNA expression in individual replicate libraries. Click here for file

Additional file 20 **Table of enriched sequence motifs identified by MEME**. Data include gPAR-CLIP crosslinking sites passing a 5% FDR threshold on target mRNAs of known RBPs identified by RIP-Chip. Click here for file

## References

[B1] Martin KC Ephrussi A mRNA localization: gene expression in the spatial dimension. Cell 2009 14 719 730 10.1016/j.cell.2009.01.044 19239891 PMC2819924

[B2] Moore MJ Proudfoot NJ Pre-mRNA processing reaches back to transcription and ahead to translation. Cell 2009 14 688 700 10.1016/j.cell.2009.02.001 19239889

[B3] Glisovic T Bachorik JL Yong J Dreyfuss G RNA-binding proteins and post-transcriptional gene regulation. FEBS Lett 2008 14 1977 1986 10.1016/j.febslet.2008.03.004 18342629 PMC2858862

[B4] Hafidh S Capkova V Honys D Safe keeping the message: mRNP complexes tweaking after transcription. Adv Exp Med Biol 2011 14 118 136 10.1007/978-1-4614-0332-6_8 21915786

[B5] Anderson P Kedersha N RNA granules: post-transcriptional and epigenetic modulators of gene expression. Nat Rev Mol Cell Biol 2009 14 430 436 19461665 10.1038/nrm2694

[B6] Lecuyer E Yoshida H Parthasarathy N Alm C Babak T Cerovina T Hughes TR Tomancak P Krause HM Global analysis of mRNA localization reveals a prominent role in organizing cellular architecture and function. Cell 2007 14 174 187 10.1016/j.cell.2007.08.003 17923096

[B7] Kato M Han TW Xie S Shi K Du X Wu LC Mirzaei H Goldsmith EJ Longgood J Pei J Grishin NV Frantz DE Schneider JW Chen S Li L Sawaya MR Eisenberg D Tycko R McKnight SL Cell-free formation of RNA granules: low complexity sequence domains form dynamic fibers within hydrogels. Cell 2012 14 753 767 10.1016/j.cell.2012.04.017 22579281 PMC6347373

[B8] Han TW Kato M Xie S Wu LC Mirzaei H Pei J Chen M Xie Y Allen J Xiao G McKnight SL Cell-free formation of RNA granules: bound RNAs identify features and components of cellular assemblies. Cell 2012 14 768 779 10.1016/j.cell.2012.04.016 22579282

[B9] Jensen KB Musunuru K Lewis HA Burley SK Darnell RB The tetranucleotide UCAY directs the specific recognition of RNA by the Nova K-homology 3 domain. Proc Natl Acad Sci USA 2000 14 5740 5745 10.1073/pnas.090553997 10811881 PMC18503

[B10] Wei WJ Mu SR Heiner M Fu X Cao LJ Gong XF Bindereif A Hui J YB-1 binds to CAUC motifs and stimulates exon inclusion by enhancing the recruitment of U2AF to weak polypyrimidine tracts. Nucleic Acids Res 2012 14 8622 8636 10.1093/nar/gks579 22730292 PMC3458536

[B11] Tenenbaum SA Carson CC Lager PJ Keene JD Identifying mRNA subsets in messenger ribonucleoprotein complexes by using cDNA arrays. Proc Natl Acad Sci USA 2000 14 14085 14090 10.1073/pnas.97.26.14085 11121017 PMC18875

[B12] Gerber AP Herschlag D Brown PO Extensive association of functionally and cytotopically related mRNAs with Puf family RNA-binding proteins in yeast. PLoS Biol 2004 14 E79 10.1371/journal.pbio.0020079 15024427 PMC368173

[B13] Hogan DJ Riordan DP Gerber AP Herschlag D Brown PO Diverse RNA-binding proteins interact with functionally related sets of RNAs, suggesting an extensive regulatory system. PLoS Biol 2008 14 e255 10.1371/journal.pbio.0060255 18959479 PMC2573929

[B14] Riordan DP Herschlag D Brown PO Identification of RNA recognition elements in the Saccharomyces cerevisiae transcriptome. Nucleic Acids Res 2010 14 1501 1509 20959291 10.1093/nar/gkq920 PMC3045596

[B15] Mili S Steitz JA Evidence for reassociation of RNA-binding proteins after cell lysis: implications for the interpretation of immunoprecipitation analyses. RNA 2004 14 1692 1694 10.1261/rna.7151404 15388877 PMC1370654

[B16] Darnell JC Mostovetsky O Darnell RB FMRP RNA targets: identification and validation. Genes Brain Behav 2005 14 341 349 10.1111/j.1601-183X.2005.00144.x 16098133

[B17] Licatalosi DD Mele A Fak JJ Ule J Kayikci M Chi SW Clark TA Schweitzer AC Blume JE Wang X Darnell JC Darnell RB HITS-CLIP yields genome-wide insights into brain alternative RNA processing. Nature 2008 14 464 469 10.1038/nature07488 18978773 PMC2597294

[B18] Ule J Jensen KB Ruggiu M Mele A Ule A Darnell RB CLIP identifies Nova-regulated RNA networks in the brain. Science 2003 14 1212 1215 10.1126/science.1090095 14615540

[B19] Ule J Jensen K Mele A Darnell RB CLIP: a method for identifying protein-RNA interaction sites in living cells. Methods 2005 14 376 386 10.1016/j.ymeth.2005.07.018 16314267

[B20] Hafner M Landthaler M Burger L Khorshid M Hausser J Berninger P Rothballer A Ascano M Jungkamp AC Munschauer M Ulrich A Wardle GS Dewell S Zavolan M Tucshl T Transcriptome-wide identification of RNA-binding protein and microRNA target sites by PAR-CLIP. Cell 2010 14 129 141 10.1016/j.cell.2010.03.009 20371350 PMC2861495

[B21] Chi SW Zang JB Mele A Darnell RB Argonaute HITS-CLIP decodes microRNA-mRNA interaction maps. Nature 2009 14 479 486 19536157 10.1038/nature08170 PMC2733940

[B22] Zisoulis DG Lovci MT Wilbert ML Hutt KR Liang TY Pasquinelli AE Yeo GW Comprehensive discovery of endogenous Argonaute binding sites in Caenorhabditis elegans. Nat Struct Mol Biol 2010 14 173 179 10.1038/nsmb.1745 20062054 PMC2834287

[B23] Mukherjee N Corcoran DL Nusbaum JD Reid DW Georgiev S Hafner M Ascano M Tuschl T Ohler U Keene JD Integrative regulatory mapping indicates that the RNA-binding protein HuR couples pre-mRNA processing and mRNA stability. Mol Cell 2011 14 327 339 10.1016/j.molcel.2011.06.007 21723170 PMC3220597

[B24] Lebedeva S Jens M Theil K Schwanhausser B Selbach M Landthaler M Rajewsky N Transcriptome-wide analysis of regulatory interactions of the RNA-binding protein HuR. Mol Cell 2011 14 340 352 10.1016/j.molcel.2011.06.008 21723171

[B25] Creamer TJ Darby MM Jamonnak N Schaughency P Hao H Wheelan SJ Corden JL Transcriptome-wide binding sites for components of the Saccharomyces cerevisiae non-poly(A) termination pathway: Nrd1, Nab3, and Sen1. PLoS Genet 2011 14 e1002329 10.1371/journal.pgen.1002329 22028667 PMC3197677

[B26] Castello A Fischer B Eichelbaum K Horos R Beckmann BM Strein C Davey NE Humphreys DT Preiss T Steinmetz LM Krijgsveld J Hentze MW Insights into RNA biology from an Atlas of mmRNA-binding proteins. Cell 2012 14 1393 1406 10.1016/j.cell.2012.04.031 22658674

[B27] Baltz AG Munschauer M Schwanhausser B Vasile A Murakawa Y Schueler M Youngs N Penfold-Brown D Drew K Milek M Wyler E Bonneau R Selbach M Dieterich C Landthaler M The mRNA-bound proteome and its global occupancy profile on protein-coding transcripts. Mol Cell 2012 14 674 690 10.1016/j.molcel.2012.05.021 22681889

[B28] Sugimoto Y Konig J Hussain S Zupan B Curk T Frye M Ule J Analysis of CLIP and iCLIP methods for nucleotide-resolution studies of protein-RNA interactions. Genome Biol 2012 14 R67 10.1186/gb-2012-13-8-r67 22863408 PMC4053741

[B29] Olivas W Parker R The Puf3 protein is a transcript-specific regulator of mRNA degradation in yeast. EMBO J 2000 14 6602 6611 10.1093/emboj/19.23.6602 11101532 PMC305854

[B30] Jackson JS Houshmandi SS Lopez Leban F Olivas WM Recruitment of the Puf3 protein to its mRNA target for regulation of mRNA decay in yeast. RNA 2004 14 1625 1636 10.1261/rna.7270204 15337848 PMC1370648

[B31] Valley CT Porter DF Qiu C Campbell ZT Hall TM Wickens M Patterns and plasticity in RNA-protein interactions enable recruitment of multiple proteins through a single site. Proc Natl Acad Sci USA 2012 14 6054 6059 10.1073/pnas.1200521109 22467831 PMC3341033

[B32] Ashe MP De Long SK Sachs AB Glucose depletion rapidly inhibits translation initiation in yeast. Mol Biol Cell 2000 14 833 848 10712503 10.1091/mbc.11.3.833 PMC14814

[B33] Gallego C Gari E Colomina N Herrero E Aldea M The Cln3 cyclin is down-regulated by translational repression and degradation during the G1 arrest caused by nitrogen deprivation in budding yeast. EMBO J 1997 14 7196 7206 10.1093/emboj/16.23.7196 9384596 PMC1170320

[B34] Simpson CE Ashe MP Adaptation to stress in yeast: to translate or not? Biochem Soc Trans 2012 14 794 799 10.1042/BST20120078 22817736

[B35] Berglund JA Chua K Abovich N Reed R Rosbash M The splicing factor BBP interacts specifically with the pre-mRNA branchpoint sequence UACUAAC. Cell 1997 14 781 787 10.1016/S0092-8674(00)80261-5 9182766

[B36] Garrey SM Voelker R Berglund JA An extended RNA binding site for the yeast branch point-binding protein and the role of its zinc knuckle domains in RNA binding. J Biol Chem 2006 14 27443 27453 10.1074/jbc.M603137200 16861232

[B37] Joo YJ Kim JH Kang UB Yu MH Kim J Gcn4p-mediated transcriptional repression of ribosomal protein genes under amino-acid starvation. EMBO J 2011 14 859 872 10.1038/emboj.2010.332 21183953 PMC3049204

[B38] Ozsolak F Kapranov P Foissac S Kim SW Fishilevich E Monaghan AP John B Milos PM Comprehensive polyadenylation site maps in yeast and human reveal pervasive alternative polyadenylation. Cell 2010 14 1018 1029 10.1016/j.cell.2010.11.020 21145465 PMC3022516

[B39] Siepel A Bejerano G Pedersen JS Hinrichs AS Hou M Rosenbloom K Clawson H Spieth J Hillier LW Richards S Weinstock GM Wilson RK Gibbs RA Kent WJ Miller W Haussler D Evolutionarily conserved elements in vertebrate, insect, worm, and yeast genomes. Genome Res 2005 14 1034 1050 10.1101/gr.3715005 16024819 PMC1182216

[B40] Chartrand P Meng XH Singer RH Long RM Structural elements required for the localization of ASH1 mRNA and of a green fluorescent protein reporter particle in vivo. Curr Biol 1999 14 333 336 10.1016/S0960-9822(99)80144-4 10209102

[B41] Gonzalez I Buonomo SB Nasmyth K von Ahsen U ASH1 mRNA localization in yeast involves multiple secondary structural elements and Ash1 protein translation. Curr Biol 1999 14 337 340 10.1016/S0960-9822(99)80145-6 10209099

[B42] Bernhart SH Hofacker IL Stadler PF Local RNA base pairing probabilities in large sequences. Bioinformatics 2006 14 614 615 10.1093/bioinformatics/btk014 16368769

[B43] Washietl S Hofacker IL Stadler PF Fast and reliable prediction of noncoding RNAs. Proc Natl Acad Sci USA 2005 14 2454 2459 10.1073/pnas.0409169102 15665081 PMC548974

[B44] Steigele S Huber W Stocsits C Stadler PF Nieselt K Comparative analysis of structured RNAs in S. cerevisiae indicates a multitude of different functions. BMC Biol 2007 14 25 10.1186/1741-7007-5-25 17577407 PMC1914338

[B45] Hohmann S Mager WH Yeast Stress Responses 2003 Revised Berlin; New York: Springer

[B46] Larochelle M Drouin S Robert F Turcotte B Oxidative stress-activated zinc cluster protein Stb5 has dual activator/repressor functions required for pentose phosphate pathway regulation and NADPH production. Mol Cell Biol 2006 14 6690 6701 10.1128/MCB.02450-05 16914749 PMC1592823

[B47] Vyas VK Berkey CD Miyao T Carlson M Repressors Nrg1 and Nrg2 regulate a set of stress-responsive genes in Saccharomyces cerevisiae. Eukaryot Cell 2005 14 1882 1891 10.1128/EC.4.11.1882-1891.2005 16278455 PMC1287862

[B48] Van Dyke N Baby J Van Dyke MW Stm1p, a ribosome-associated protein, is important for protein synthesis in Saccharomyces cerevisiae under nutritional stress conditions. J Mol Biol 2006 14 1023 1031 10.1016/j.jmb.2006.03.018 16580682

[B49] Griac P Henry SA The yeast inositol-sensitive upstream activating sequence, UASINO, responds to nitrogen availability. Nucleic Acids Res 1999 14 2043 2050 10.1093/nar/27.9.2043 10198439 PMC148419

[B50] Schreve JL Garrett JM Yeast Agp2p and Agp3p function as amino acid permeases in poor nutrient conditions. Biochem Biophys Res Commun 2004 14 745 751 10.1016/j.bbrc.2003.11.172 14697254

[B51] Grousl T Ivanov P Frydlova I Vasicova P Janda F Vojtova J Malinska K Malcova I Novakova L Janoskova D Valasek L Hasek J Robust heat shock induces eIF2alpha-phosphorylation-independent assembly of stress granules containing eIF3 and 40S ribosomal subunits in budding yeast, Saccharomyces cerevisiae. J Cell Sci 2009 14 2078 2088 10.1242/jcs.045104 19470581

[B52] Dreyfuss G Kim VN Kataoka N Messenger-RNA-binding proteins and the messages they carry. Nat Rev Mol Cell Biol 2002 14 195 205 10.1038/nrm760 11994740

[B53] Balagopal V Parker R Polysomes, P bodies and stress granules: states and fates of eukaryotic mRNAs. Curr Opin Cell Biol 2009 14 403 408 10.1016/j.ceb.2009.03.005 19394210 PMC2740377

[B54] Rogelj B Easton LE Bogu GK Stanton LW Rot G Curk T Zupan B Sugimoto Y Modic M Haberman N Tollervey J Fujii R Takumi T Shaw CE Ule J Widespread binding of FUS along nascent RNA regulates alternative splicing in the brain. Sci Rep 2012 14 603 22934129 10.1038/srep00603 PMC3429604

[B55] Ghaemmaghami S Huh WK Bower K Howson RW Belle A Dephoure N O'Shea EK Weissman JS Global analysis of protein expression in yeast. Nature 2003 14 737 741 10.1038/nature02046 14562106

[B56] Favre A Moreno G Salet C Vinzens F 4-Thiouridine incorporation into the RNA of monkey kidney cells (CV-1) triggers near-UV light long-term inhibition of DNA, RNA and protein synthesis. Photochem Photobiol 1993 14 689 694 10.1111/j.1751-1097.1993.tb04953.x 7506834

[B57] Levin JZ Yassour M Adiconis X Nusbaum C Thompson DA Friedman N Gnirke A Regev A Comprehensive comparative analysis of strand-specific RNA sequencing methods. Nat Methods 2010 14 709 715 10.1038/nmeth.1491 20711195 PMC3005310

[B58] Langmead B Trapnell C Pop M Salzberg SL Ultrafast and memory-efficient alignment of short DNA sequences to the human genome. Genome Biol 2009 14 R25 10.1186/gb-2009-10-3-r25 19261174 PMC2690996

[B59] Yassour M Kaplan T Fraser HB Levin JZ Pfiffner J Adiconis X Schroth G Luo S Khrebtukova I Gnirke A Nusbaum C Thompson DA Friedman N Regev A Ab initio construction of a eukaryotic transcriptome by massively parallel mRNA sequencing. Proc Natl Acad Sci USA 2009 14 3264 3269 10.1073/pnas.0812841106 19208812 PMC2638735

[B60] Nagalakshmi U Wang Z Waern K Shou C Raha D Gerstein M Snyder M The transcriptional landscape of the yeast genome defined by RNA sequencing. Science 2008 14 1344 1349 10.1126/science.1158441 18451266 PMC2951732

[B61] Hofacker IL Fontana W Stadler PF Bonhoeffer LS Tacker M Schuster P Fast Folding and Comparison of Rna Secondary Structures. Monatshefte Fur Chemie 1994 14 167 188 10.1007/BF00818163

[B62] Darty K Denise A Ponty Y VARNA: Interactive drawing and editing of the RNA secondary structure. Bioinformatics 2009 14 1974 1975 10.1093/bioinformatics/btp250 19398448 PMC2712331

[B63] Bailey TL Elkan C Fitting a mixture model by expectation maximization to discover motifs in biopolymers. Proc Int Conf Intell Syst Mol Biol 1994 14 28 36 7584402

[B64] Alexa A Rahnenfuhrer J Lengauer T Improved scoring of functional groups from gene expression data by decorrelating GO graph structure. Bioinformatics 2006 14 1600 1607 10.1093/bioinformatics/btl140 16606683

[B65] Gene Expression Omnibus http://www.ncbi.nlm.nih.gov/geo/

