# Supplementary material for: Pervasive and dynamic protein binding sites of the mRNA transcriptome in Saccharomyces cerevisiae
Source: Genome Biol. 2013 Feb 14;14(2):R13. doi: 10.1186/gb-2013-14-2-r13 (PMC4053964; doi:10.1186/gb-2013-14-2-r13)
Supplement: Additional file 1 — Sequencing and mapping statistics. Read counts and T-to-C conversion rates for all gPAR-CLIP, mRNA-seq, and PAR-CLIP libraries. [file gb-2013-14-2-r13-S1.PDF]

Additional file 1

condition	*protocol*	*4sU added?*	*Ribosome depletion?*	*total raw reads*	*total high quality reads*	*total mapped reads*			*total mapped reads (1 T>C)*	*% of high quality reads*	*total mapped reads (2 T>C)*	*% of high quality reads*
						0 mm	*1 mm*	*2 mm*				
WT	gPAR-CLIP	Yes	Yes	10,345,660	9,892,996	855,723	5,652,529	1,080,794	5,396,767	54.6%	692,138	7.0%
WT	gPAR-CLIP	Yes	Yes	13,354,315	12,354,153	1,020,368	7,138,708	1,184,089	6,848,424	55.4%	728,086	5.9%
-glucose	gPAR-CLIP	Yes	Yes	8,698,657	8,571,862	1,186,644	4,130,861	683,064	3,808,408	44.4%	275,737	3.2%
-glucose	gPAR-CLIP	Yes	Yes	11,545,105	11,415,553	1,587,380	5,253,564	973,283	4,828,045	42.3%	400,089	3.5%
-nitrogen	gPAR-CLIP	Yes	Yes	12,565,700	12,315,329	1,846,036	6,128,110	837,766	5,654,249	45.9%	274,345	2.2%
-nitrogen	gPAR-CLIP	Yes	Yes	7,524,557	7,393,201	1,009,996	3,616,734	508,681	3,334,758	45.1%	174,574	2.4%
WT	mRNA-seq	Yes	No	11,164,331	11,126,176	8,074,185	823,549	92,132	341,800	3.1%	9,700	0.1%
WT	mRNA-seq	Yes	No	11,626,588	11,596,323	8,495,022	888,394	105,960	345,756	3.0%	9,539	0.1%
WT	mRNA-seq	Yes	Yes	20,881,199	20,881,199	15,528,464	161,166	119,230	47,856	0.2%	2,241	0.0%
WT	mRNA-seq	Yes	Yes	19,254,100	19,254,100	14,955,841	145,716	110,676	47,400	0.2%	3,927	0.0%
WT	mRNA-seq	No	Yes	18,570,981	18,570,981	14,911,218	206,401	157,157	34,727	0.2%	2,567	0.0%
WT	mRNA-seq	No	Yes	18,618,595	18,618,595	14,654,128	140,281	112,501	22,707	0.1%	2,185	0.0%
-glucose	mRNA-seq	Yes	Yes	20,764,203	20,764,203	17,367,447	143,677	114,825	26,350	0.1%	2,251	0.0%
-glucose	mRNA-seq	Yes	Yes	16,396,102	16,396,102	13,415,811	115,996	92,410	22,998	0.1%	2,518	0.0%
-nitrogen	mRNA-seq	Yes	Yes	17,424,942	17,424,942	14,094,672	145,566	102,798	31,929	0.2%	2,246	0.0%
-nitrogen	mRNA-seq	Yes	Yes	24,514,654	24,514,654	19,641,672	220,397	157,438	42,438	0.2%	3,057	0.0%
WT (Puf3p IP)	PAR-CLIP	Yes	No	10,498,429	8,394,537	1,450,253	3,431,211	470,044	3,219,759	38.4%	181,296	2.2%
