# Supplementary figures and images for: Pervasive and dynamic protein binding sites of the mRNA transcriptome in Saccharomyces cerevisiae
Source: Genome Biol. 2013 Feb 14;14(2):R13. doi: 10.1186/gb-2013-14-2-r13 (PMC4053964; doi:10.1186/gb-2013-14-2-r13)

Additional File 2

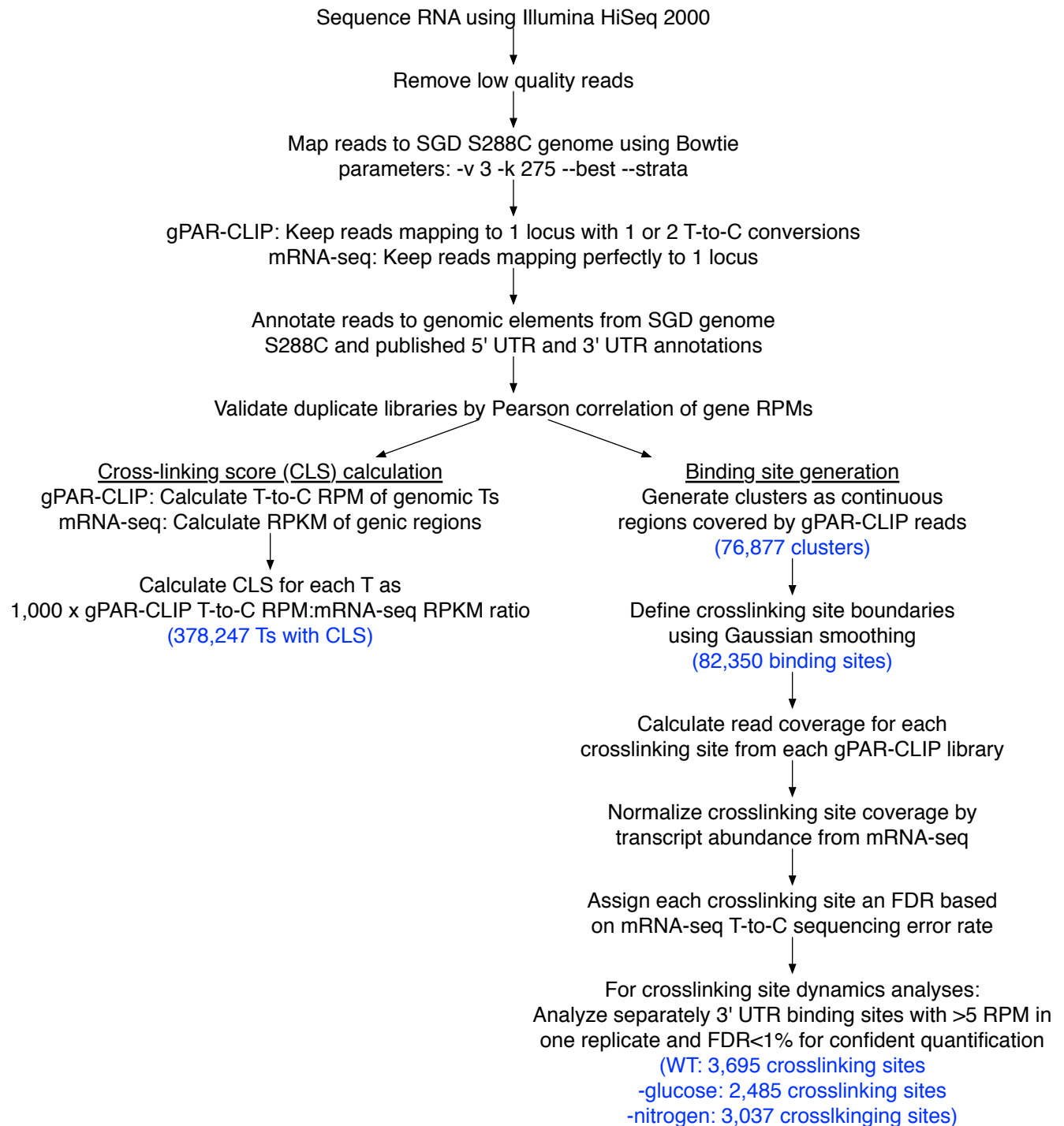

Supplement: Additional file 2 — Pipeline for generating crosslinking scores and crosslinking sites. Processing steps used to generate crosslinking scores and crosslinking sites from gPAR-CLIP and mRNA-seq data. [file gb-2013-14-2-r13-S2.PDF]

Additional File 4

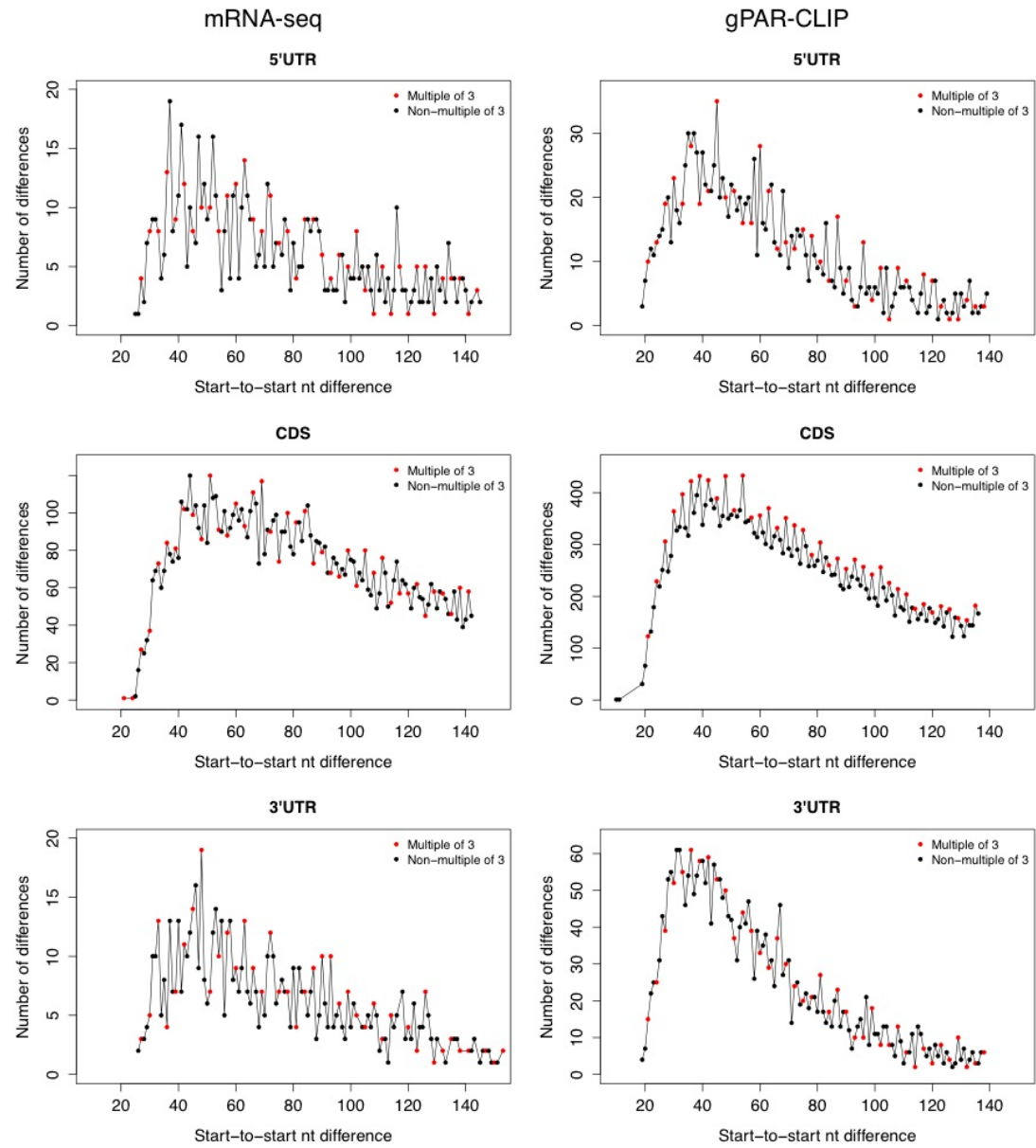

Supplement: Additional file 5 — Visualization of crosslinking site periodicity. Distribution of start-to-start nucleotide distances between 5' UTR, CDS, and 3' UTR read clusters from gPAR-CLIP and mRNA-seq libraries. Only distances from gPAR-CLIP CDS read clusters were enriched for multiples of 3 (red dots). [file gb-2013-14-2-r13-S5.PDF]

Additional File 5

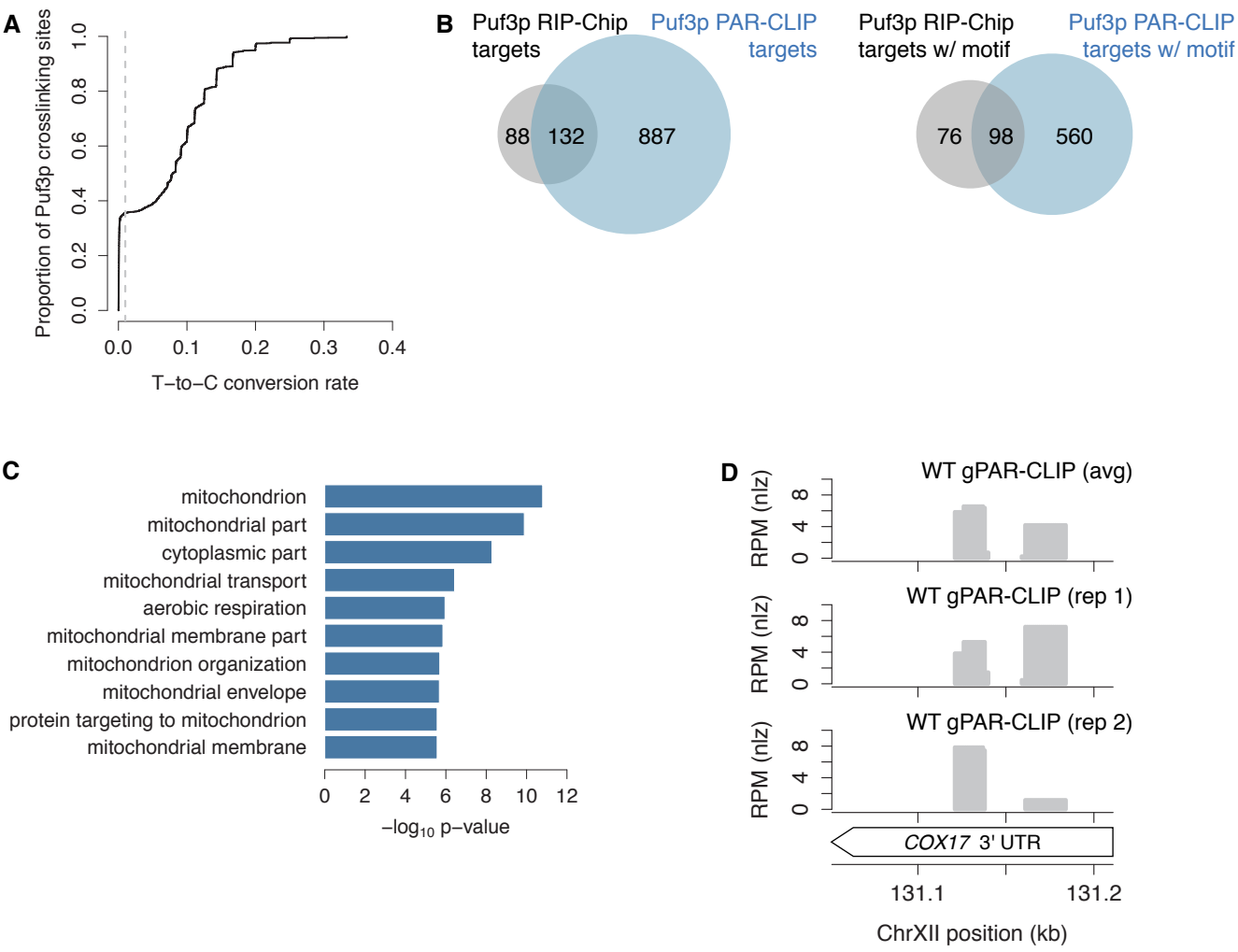

Supplement: Additional file 8 — Analysis and comparison of PAR-CLIP-identified Puf3p targets. (A) Puf3p PAR-CLIP identified crosslinking sites in 147 (67%) of the 220 Puf3p target mRNA identified by RIP-Chip; 174 Puf3p RIP-Chip-identified target mRNAs contain the Puf3p recognition motif UGUAAAUA [12,13]. Puf3p PAR-CLIP identified motif-containing crosslinking sites in 76 (44%) of these mRNAs and in 265 additional mRNAs, suggesting post-transcriptional regulation by Puf3p for these 265 novel targets. (B) GO enrichment analysis of 265 PAR-CLIP-identified, motif-containing Puf3p targets. Results are consistent with Puf3p's role in localization, deadenylation, and repression of mRNAs encoding proteins destined for the mitochondria [29]. (C) Individual replicate coverage of COX17 3' UTR in gPAR-CLIP with average coverage as shown in Figure 2b. [file gb-2013-14-2-r13-S8.PDF]

Additional File 6

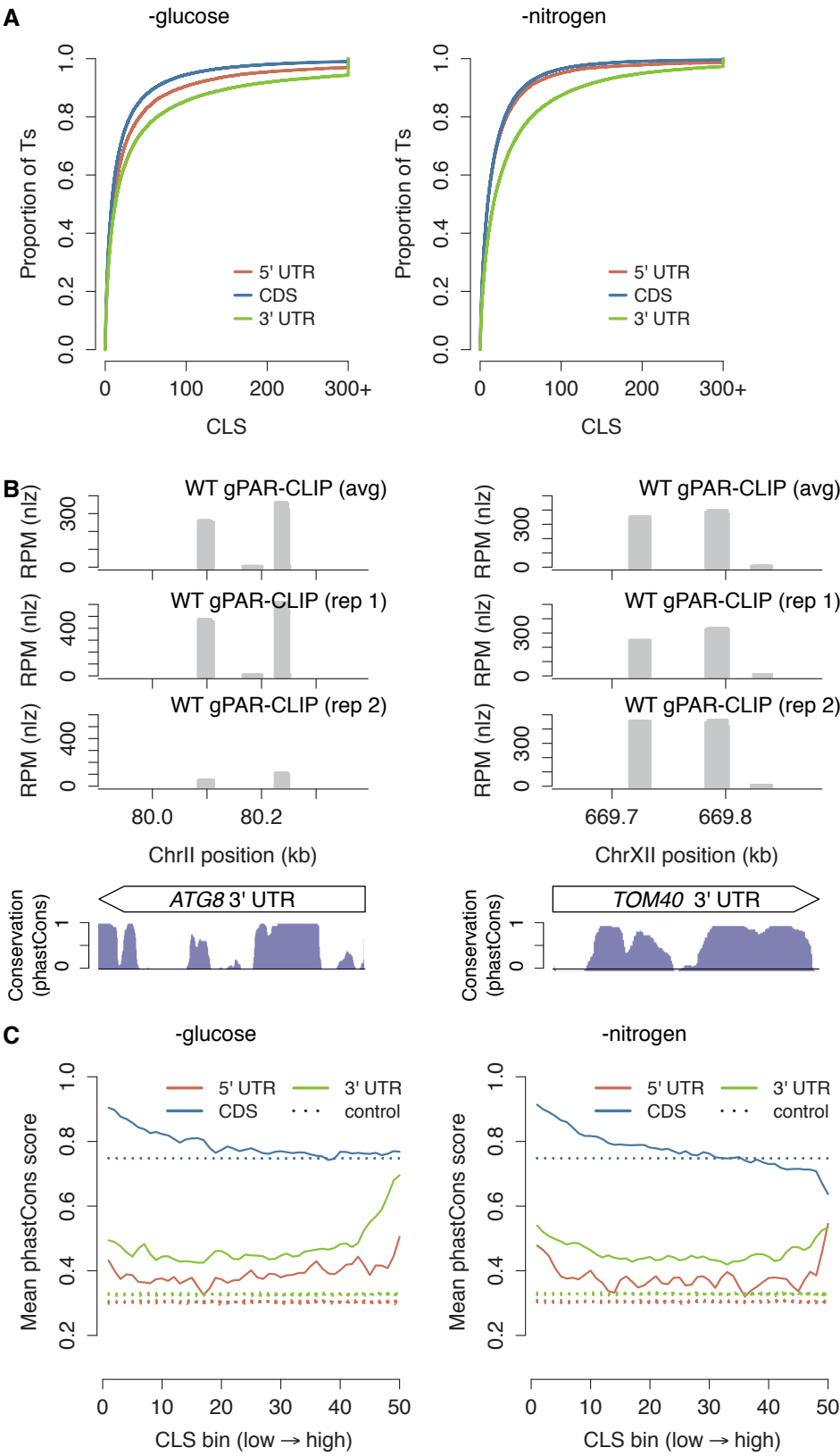

Supplement: Additional file 11 — Analysis of crosslinking scores and conservation of genomic Ts in starvation conditions. (A) Cumulative distribution of CLSs from 5' UTR, CDS, and 3' UTR regions. (B) Individual replicate coverage of ATG8 and TOM40 3' UTRs in gPAR-CLIP with average coverage as shown in Figure 3d. (C) Mean phastCons scores for Ts ranked and binned by CLSs. Control lines represent mean phastCons scores of randomly ranked and binned Ts with no CLS, repeated ten times. [file gb-2013-14-2-r13-S11.PDF]

Additional File 7

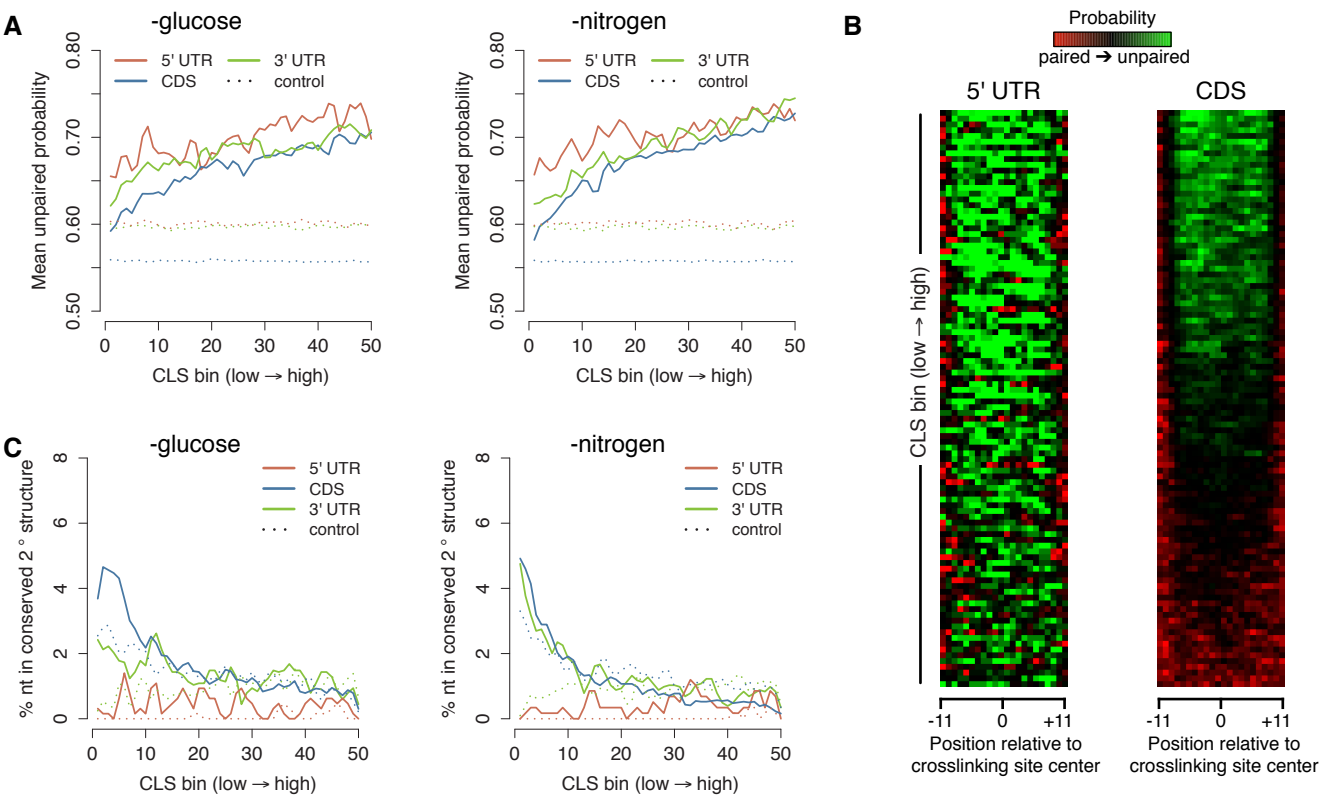

Supplement: Additional file 12 — Analysis of RNA secondary structure in starvation conditions. (A) Mean unpaired probability scores for Ts ranked and binned by CLSs. Control lines represent mean unpaired probability of randomly ranked and binned Ts with no CLS, repeated ten times. (B) Heatmaps of pairedness of 5' UTR and CDS crosslinking sites ranked by average crosslinking site CLS. (C) Percentage of Ts ranked and binned by CLSs in conserved secondary structural elements as defined by RNAz [43,44]. Control lines represent percentage of randomly ranked and binned Ts with no CLS in conserved secondary structural elements, repeated ten times. [file gb-2013-14-2-r13-S12.PDF]

# Additional File 8

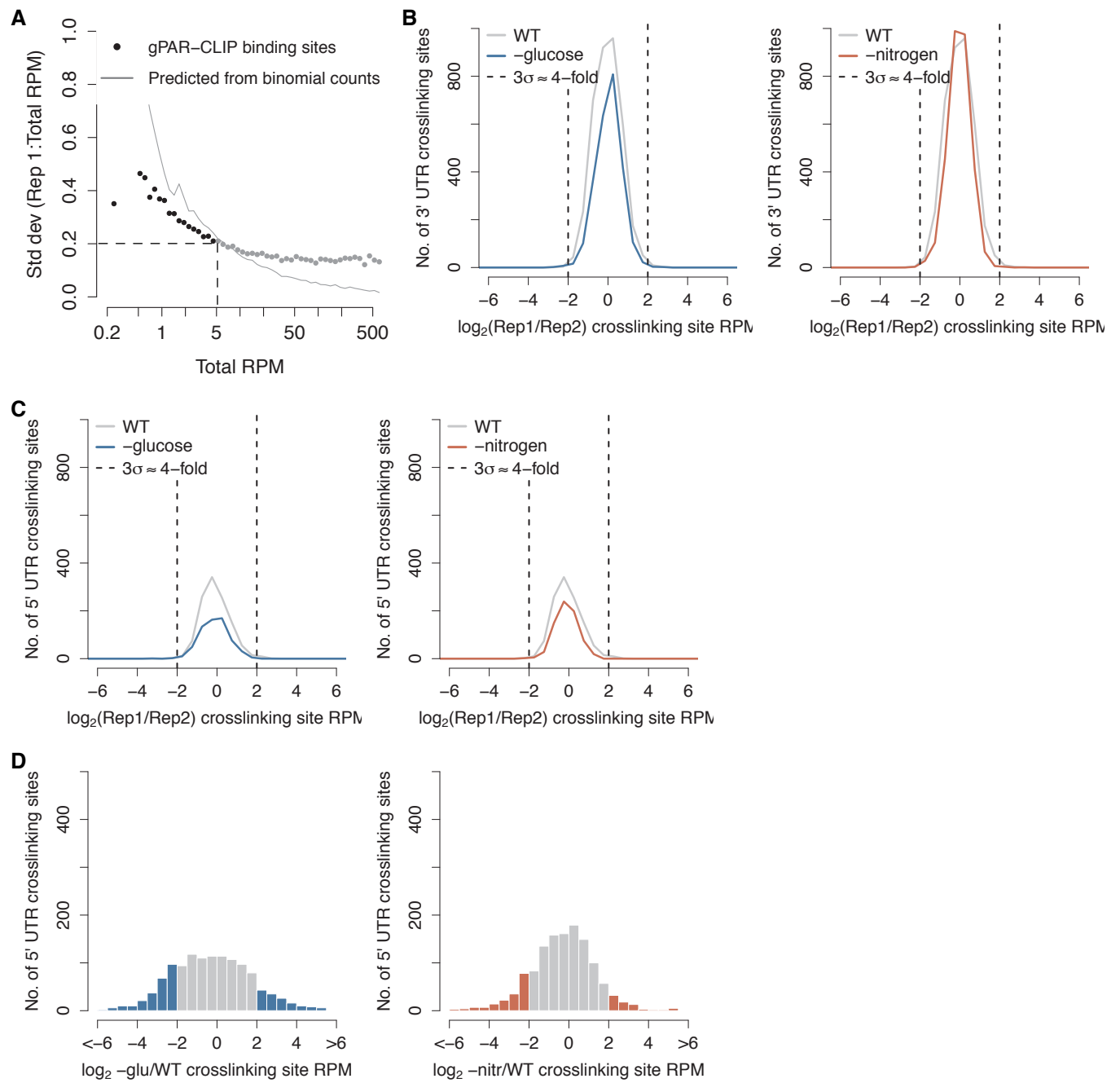

Supplement: Additional file 13 — Intra-replicate variation of crosslinking site coverage and global changes in 5' UTR crosslinking sites. (A) Determination of minimum crosslinking site coverage required for comparison of sites across environmental conditions. We chose 5 RPM as the minimum crosslinking site coverage needed for confident quantification since, at this coverage, the standard deviation of the fraction of crosslinking site reads coming from one replicate library stabilized at <0.2. Shown are data from wild-type (WT) replicate libraries; similar results were obtained for all library types. (B) Intra-replicate variation of 3' UTR crosslinking sites in WT and glucose (left) or nitrogen (right) starvation conditions. Dotted lines represent three standard deviations from the mean and correspond to approximately four-fold change between WT replicates. (C) Same as (A) but for 5' UTR crosslinking sites. (D) Global changes in 5' UTR crosslinking site coverage upon glucose (left) or nitrogen (right) starvation. [file gb-2013-14-2-r13-S13.PDF]

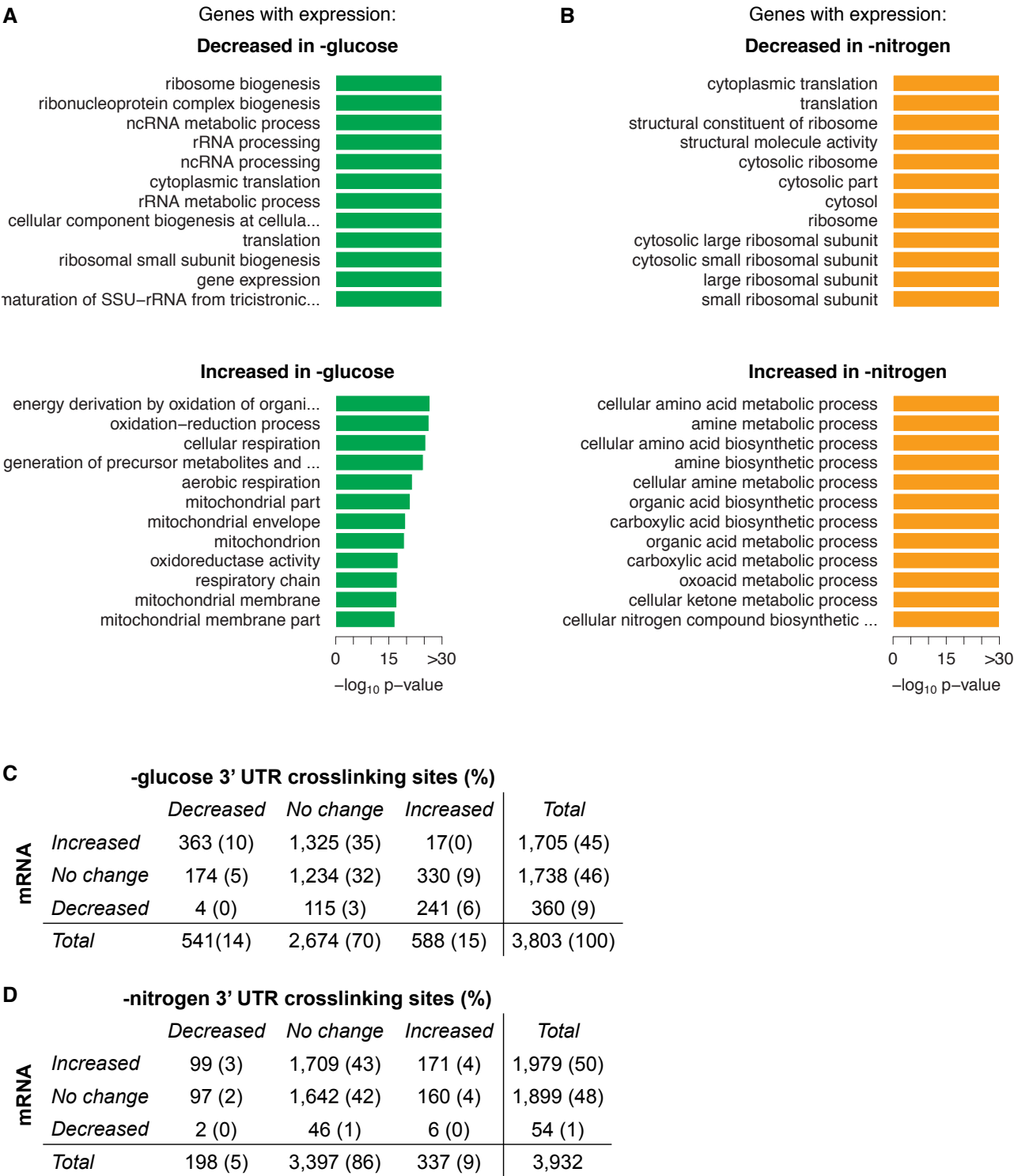

Supplement: Additional file 15 — Assessment of crosslinking site and mRNA changes in starvation conditions. (A,B) Enriched GO terms of genes up- and down-regulated upon glucose (A) or nitrogen (B) starvation. (C) The number and percentage of 3' UTR crosslinking sites with indicated changes in crosslinking site coverage and corresponding mRNA expression upon glucose starvation. (D) The number and percentage of 3' UTR crosslinking sites with indicated changes in crosslinking site coverage and corresponding mRNA expression upon nitrogen starvation. [file gb-2013-14-2-r13-S15.PDF]

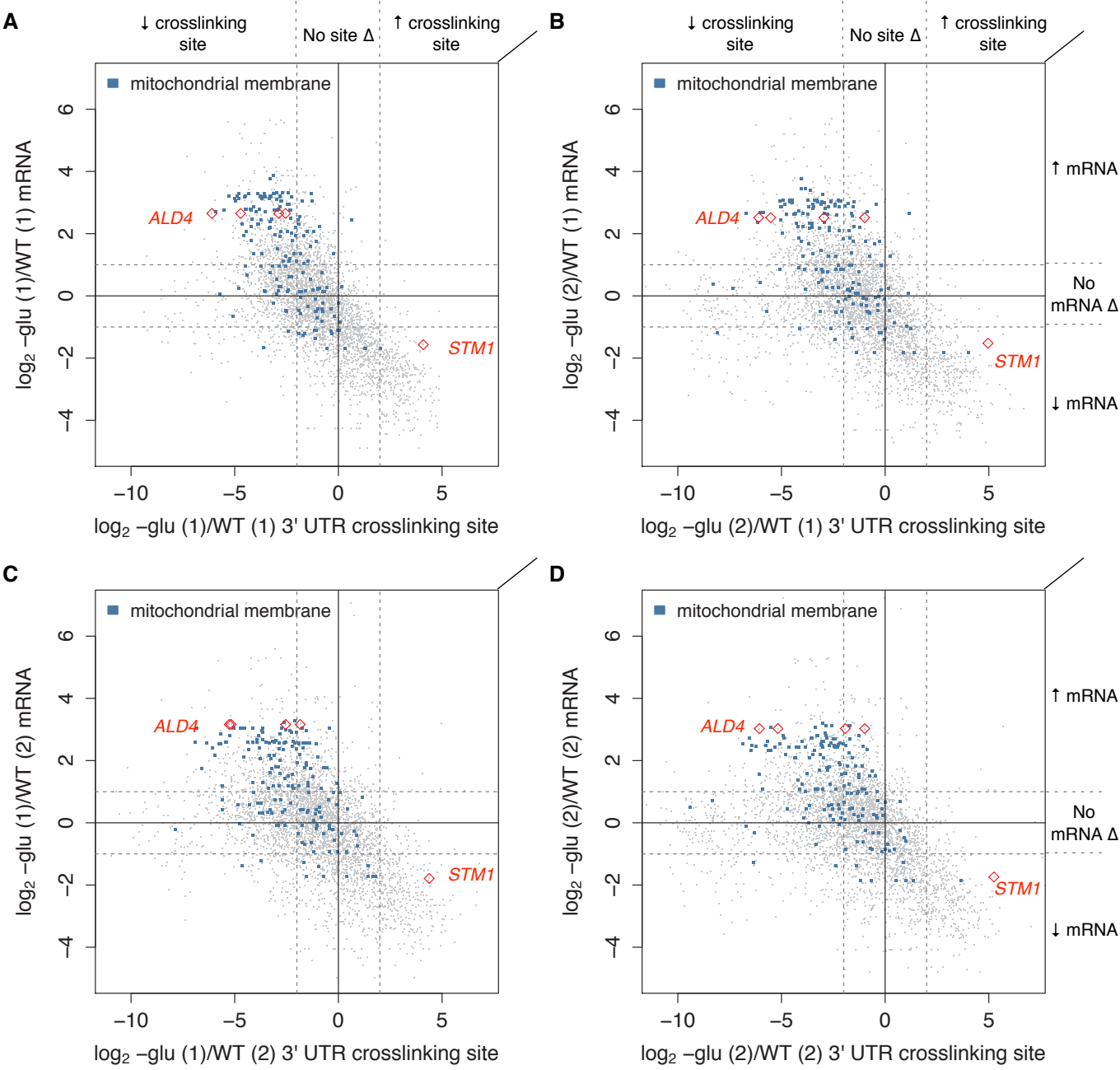

Supplement: Additional file 16 — Global changes in 3' UTR crosslinking site upon glucose starvation. Changes in crosslinking site coverage from one replicate library each of wild-type (WT) and glucose starvation conditions are plotted versus changes in the corresponding mRNA from one replicate library each of WT and glucose starvation conditions. Dotted lines and colors are as in Figure 6a. [file gb-2013-14-2-r13-S16.PDF]

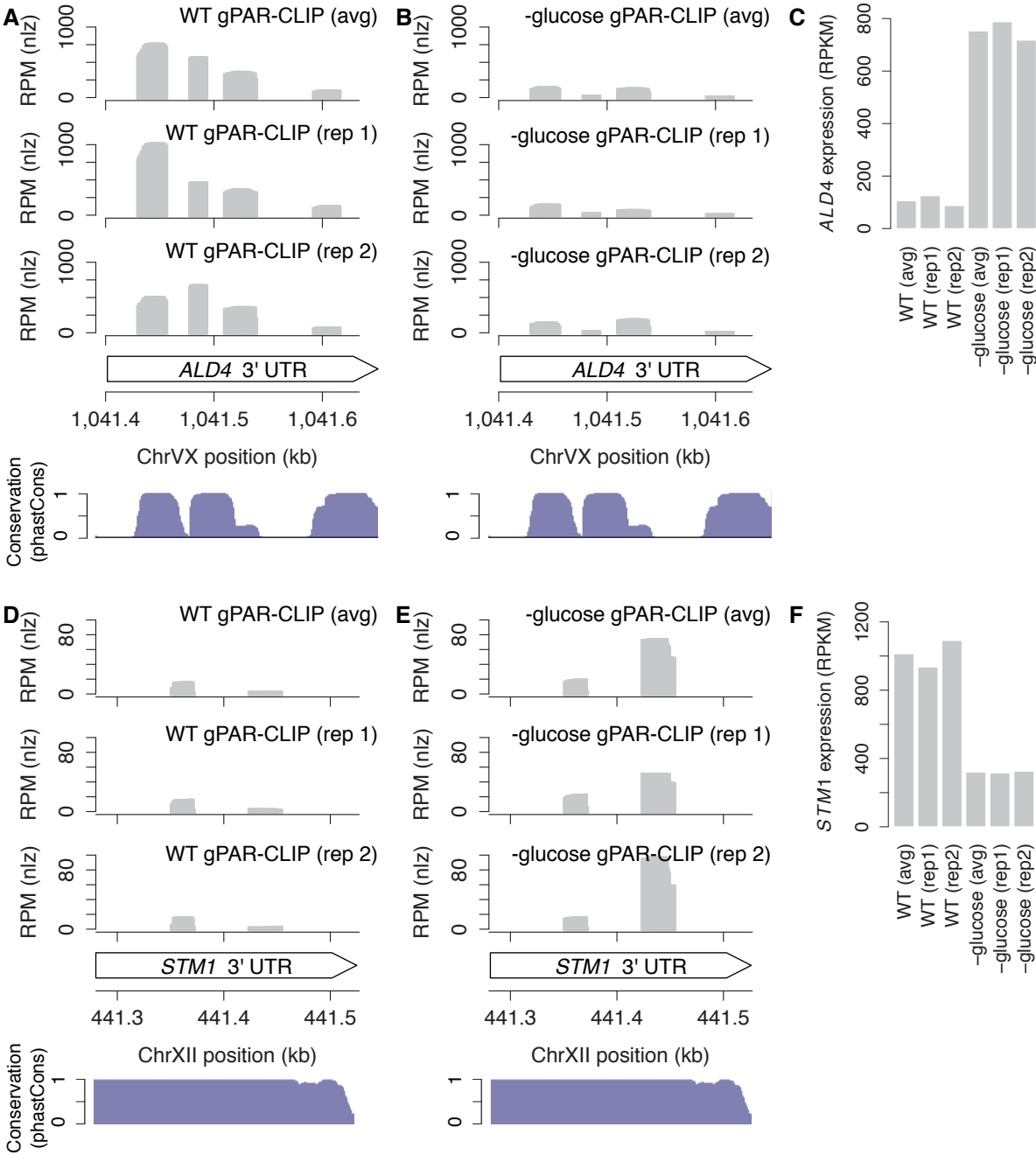

Supplement: Additional file 17 — Changes in 3' UTR crosslinking sites on ALD4 and STM1 upon glucose starvation. Same as Figure 6b,c but showing crosslinking site coverage and mRNA expression in individual replicate libraries. [file gb-2013-14-2-r13-S17.PDF]

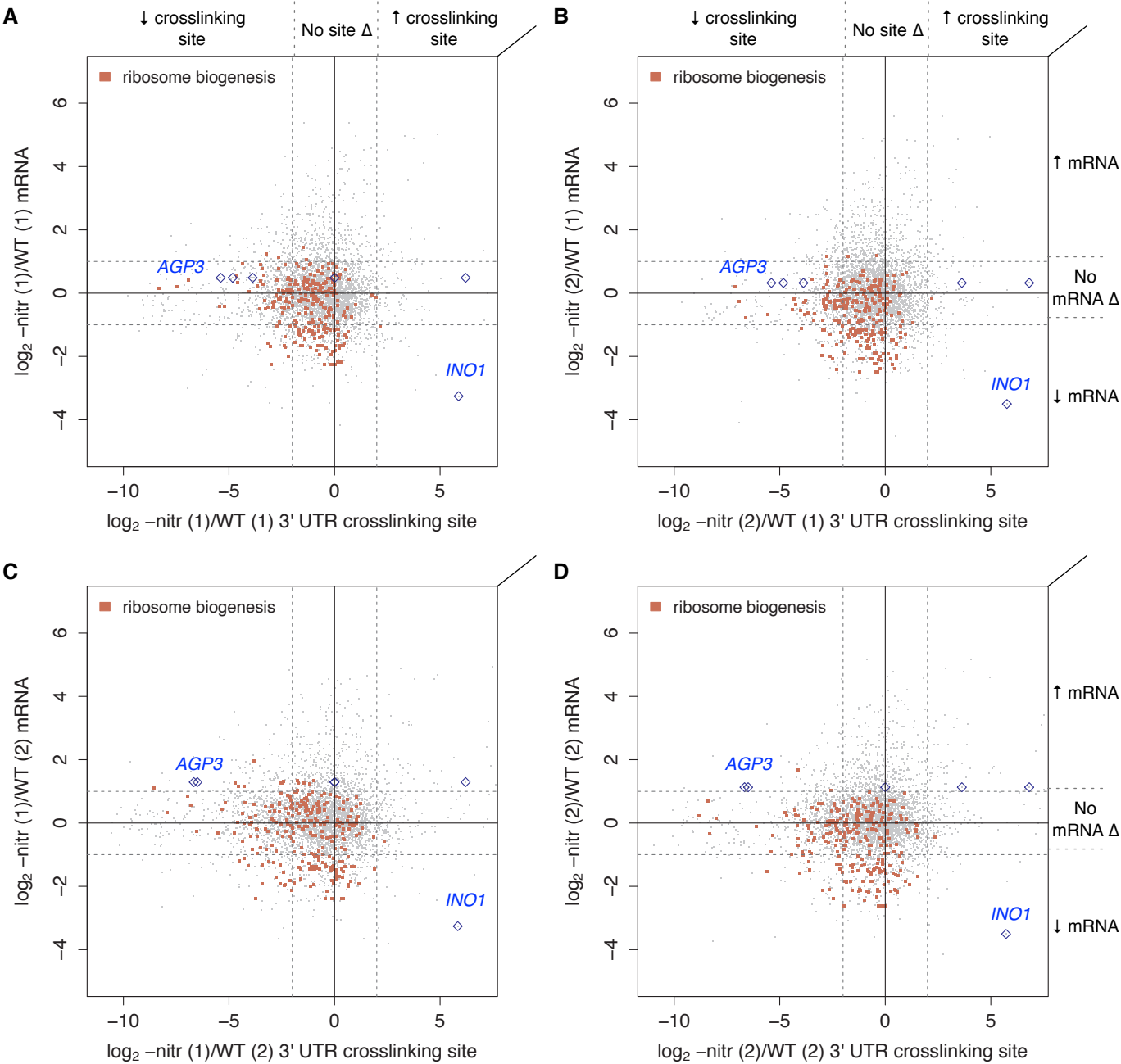

Supplement: Additional file 18 — Global changes in 3' UTR crosslinking site upon nitrogen starvation. Changes in crosslinking site coverage from one replicate library each of wild-type (WT) and nitrogen starvation conditions are plotted versus changes in the corresponding mRNA from one replicate library each of WT and nitrogen starvation conditions. Dotted lines and colors are as in Figure 7a. [file gb-2013-14-2-r13-S18.PDF]

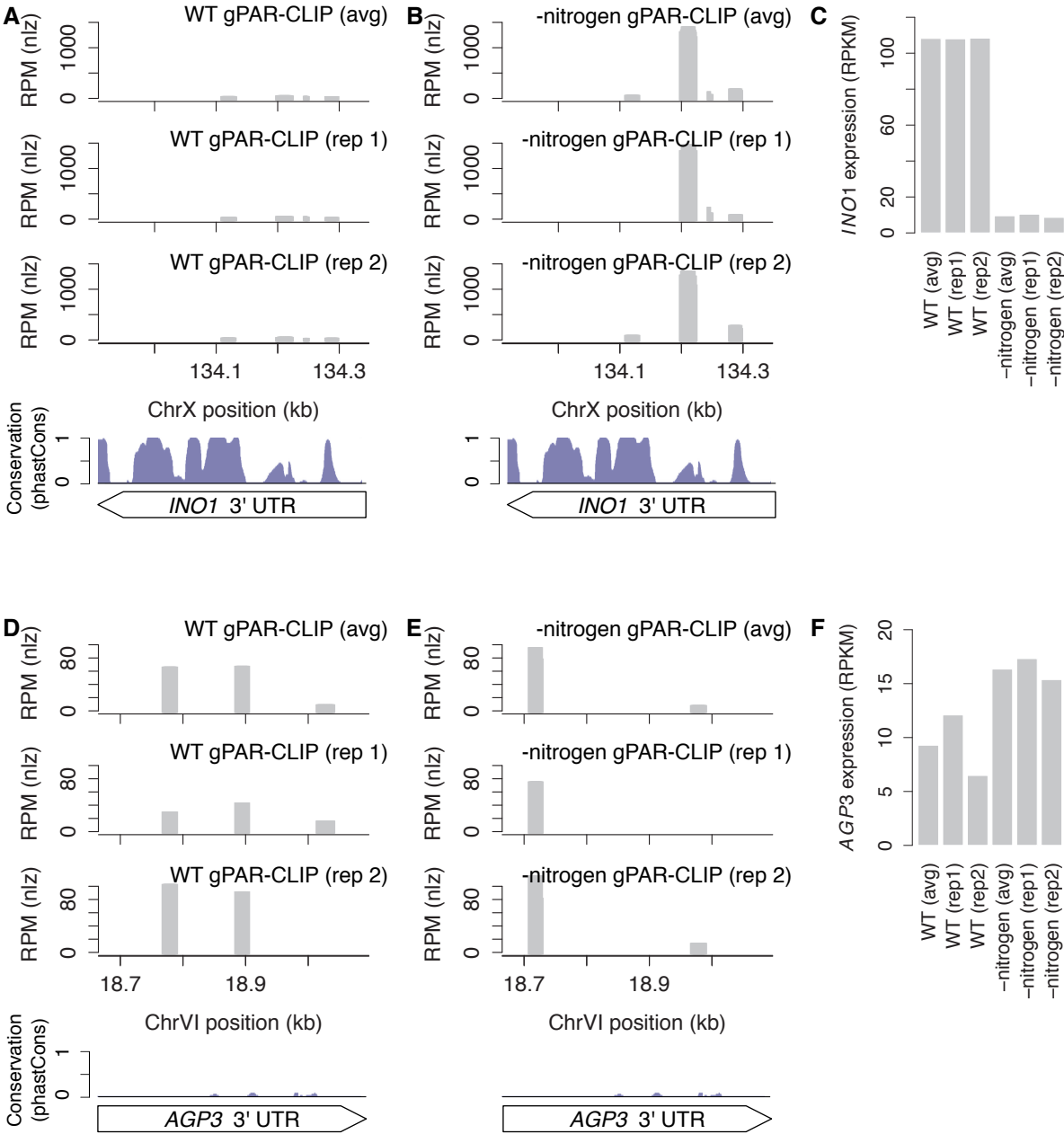

Supplement: Additional file 19 — Changes in 3' UTR crosslinking sites on INO1 and AGP3 upon glucose starvation. Same as Figure 7b,c but showing crosslinking site coverage and mRNA expression in individual replicate libraries. [file gb-2013-14-2-r13-S19.PDF]
