# Supplementary material for: Pervasive and dynamic protein binding sites of the mRNA transcriptome in Saccharomyces cerevisiae
Source: Genome Biol. 2013 Feb 14;14(2):R13. doi: 10.1186/gb-2013-14-2-r13 (PMC4053964; doi:10.1186/gb-2013-14-2-r13)

Additional File 3

A

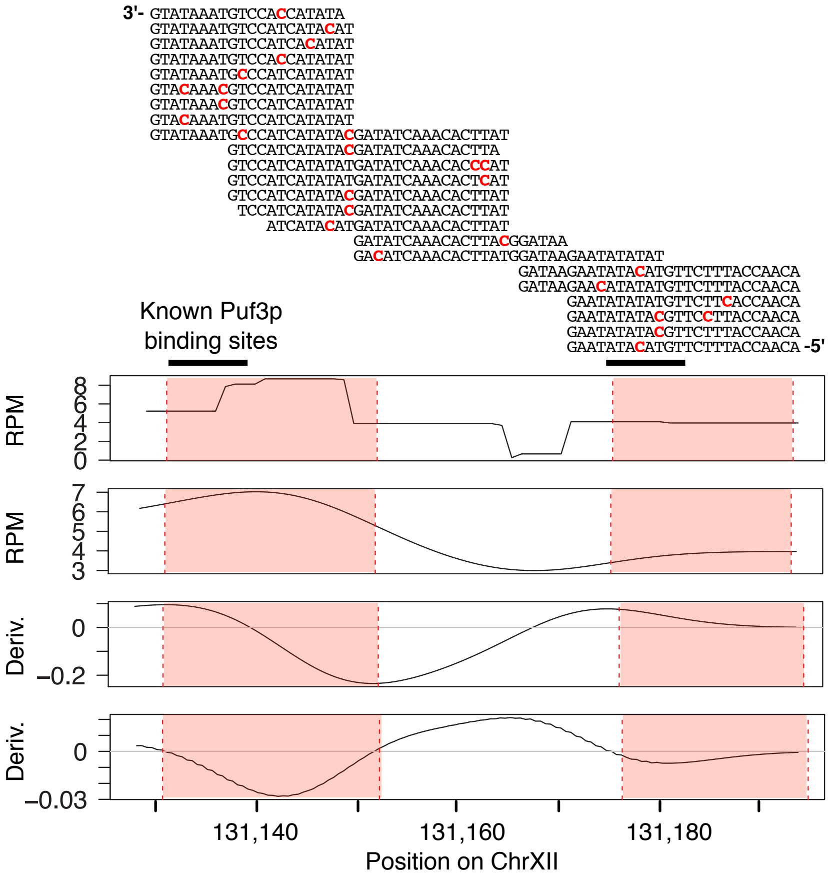

B

Library type	Pearson R^2
gPAR-CLIP replicates	
WT	0.968
-glucose	0.967
-nitrogen	0.971
mRNA-seq replicates	
WT (no 4sU)	0.988
WT	0.984
-glucose	0.994
-nitrogen	0.991
WT (no 4sU) vs. WT	0.982

C

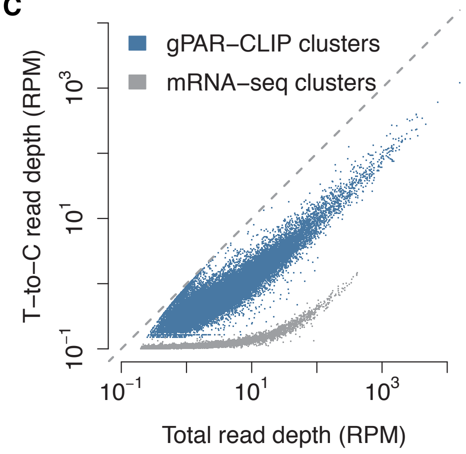

D

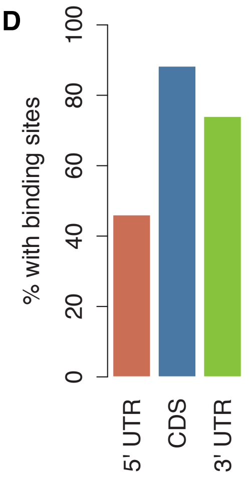

Supplement: Additional file 3 — Computational identification of crosslinking sites. (A) Illustration of sequence block generation, Gaussian distribution fitting, and cluster segmentation to identify individual crosslinking sites. (B) Pearson correlation coefficients for all gPAR-CLIP and mRNA-seq replicate libraries based on gene RPM values. (C) Separation of T-to-C sequencing errors from crosslinking-induced mismatches. Plotted for each cluster is T-to-C RPM coverage versus total RPM coverage from gPAR-CLIP or mRNA-seq libraries. (D) Percentage of annotated 5' UTR, CDS, and 3' UTR regions with at least one crosslinking site with >5 RPM. [file gb-2013-14-2-r13-S3.PDF]
